# ZIP8 Regulates Inflammation and Macrophage Polarisation in Intervertebral Disc Degeneration via the Wnt/β‐Catenin Pathway

**DOI:** 10.1111/jcmm.70431

**Published:** 2025-02-24

**Authors:** Jun Xu, Huijie Gu, Kaifeng Zhou, Liang Wu, Yiming Zhang, Chong Bian, Zhongyue Huang, Guangnan Chen, Xiangyang Cheng, Xiaofan Yin

**Affiliations:** ^1^ Department of Orthopaedics, Minhang Hospital Fudan University Shanghai China

**Keywords:** inflammation, intervertebral disc degeneration, M1 macrophage polarisation, Wnt/β‐catenin signalling pathway, *ZIP8*

## Abstract

One main cause of persistent back discomfort is intervertebral disc degeneration (IDD), with inflammation and extracellular matrix (ECM) degradation playing critical roles. This study investigates the role of *ZIP8*, a zinc transporter, in IDD pathogenesis, focusing on its effects on inflammatory responses, ECM degradation and Wnt/β‐catenin signalling pathway. *ZIP8* was identified as a hub gene from the GSE27494 dataset through bioinformatics analysis. The role of *ZIP8* was investigated in nucleus pulposus (NP) cells and RAW 264.7 macrophages. An in vivo IDD rat model was used to assess the consequences of *ZIP8* overexpression. The involvement of the Wnt/β‐catenin pathway was examined, and the effect on macrophage polarisation was analysed. *ZIP8* overexpression in NP cells led to increased inflammatory cytokine production and enhanced NF‐κB pathway activation, while *ZIP8* knockdown alleviated these effects. In vitro, *ZIP8* knockdown reduced IL‐1β–induced apoptosis and ECM degradation, promoting cell viability. In vivo, *ZIP8* overexpression exacerbated disc degeneration, as evidenced by magnetic resonance imaging (MRI) and histological assessments. Additionally, modulation of ZIP8, in conjunction with the Wnt/β‐catenin signalling pathway, revealed its involvement in regulating apoptosis and proliferation in NP cells. In RAW 264.7 macrophages, *ZIP8* knockdown inhibited M1 macrophage polarisation and reduced proinflammatory cytokine expression, while promoting anti‐inflammatory responses. *ZIP8* is a key regulator in IDD, affecting inflammation, ECM integrity and Wnt/β‐catenin signalling pathways. Targeting *ZIP8* by knockdown may offer therapeutic potential in IDD by modulating inflammatory responses and protecting ECM structure, offering a novel approach to IDD treatment.

## 1 Introduction

A progressive deterioration of the intervertebral disc, characterised by a decline in water content and the onset of fibrosis, consequently compromising disc stability, is known as intervertebral disc degeneration (IDD) [1]. Clinically, IDD often presents as diminished longitudinal elasticity with a peripheral expansion and flattening, evident as disc bulging in radiographic images [2]. While many individuals remain asymptomatic, a subset presents with clinical manifestations including low back pain, cervical discomfort or pain in the lower extremities [3, 4]. Furthermore, IDD is associated with various spinal complications, such as disc herniation, spinal stenosis and sciatica [5], which can lead to significant health issues and economic burdens. Conventional surgical and conservative interventions, although beneficial in addressing spinal complications, do not offer a definitive cure for IDD [6]. Therefore, gaining a deeper understanding of the pathogenic mechanisms of IDD and developing new therapeutic strategies hold significant clinical importance. Preliminary research has linked IDD pathogenesis to genetic predispositions, mechanical stress, inflammation and enzymatic degradation [7]. Among these factors, inflammation has become an important participant, exacerbating degeneration and influencing the beginning of pain and structural changes within the disc. Proinflammatory cytokines and mediators contribute to the breakdown of extracellular matrix (ECM) components, further destabilising the disc and perpetuating a cycle of degeneration and inflammation [8]. Despite these insights, a comprehensive understanding of the aetiology of IDD remains elusive, and further intensive research is needed to develop targeted therapies that can effectively interrupt this destructive cycle.

Macrophages are versatile immune cells critical for host defence and tissue homeostasis [9]. M1 and M2 are the two main phenotypes into which they can polarise. Macrophages stimulated by interferon‐gamma (IFN‐γ) and lipopolysaccharide (LPS), known as M1 macrophages, are proinflammatory and are pivotal in pathogen elimination and initiating inflammatory responses [10]. Conversely, macrophages activated by interleukin‐4 (IL‐4) and interleukin‐13 (IL‐13), known as M2 macrophages, are anti‐inflammatory and facilitate tissue repair and the resolution of inflammation [11]. The equilibrium between M1 and M2 polarisation is essential for sustaining immune homeostasis and effectively addressing injury and infection. Recent studies have highlighted the role of macrophage polarisation in IDD. Zhao et al. demonstrated that M1 polarisation, induced by exosomes derived from degenerated nucleus pulposus (NP) cells, exacerbates IDD through the miR‐27a‐3p pathway, targeting PPARγ/NF‐κB/PI3K/AKT signalling, thereby promoting inflammation and disc degradation [12]. Similarly, Zhao et al. reported that M1 macrophage polarisation aggravates IDD by inducing inflammation and NP cell apoptosis [13]. However, treatment with magnoflorine (MAG) mitigates this harm caused by inhibiting the HMGB1/MyD88/NF‐κB pathway and the NLRP3 inflammasome, reducing proinflammatory cytokines and apoptosis‐related proteins. These findings underscore the significant research value of macrophages in the context of IDD and suggest potential therapeutic targets for mitigating disc degeneration.

In addition to the fundamental understanding of IDD, recent studies have emphasised the significance of specific molecular transporters in various diseases. Among these, the zinc transporter *SLC39A* family, commonly referred to as the *ZIP* family, comprises 14 members primarily involved in facilitating the transport of zinc ions from the ECM or organelles into the cytoplasm [14, 15]. These transporters are pivotal for cellular zinc homeostasis [16]. The implication of the ZIP family in various pathological conditions, especially in tumorigenesis, is noteworthy. For instance, *ZIP1* and *ZIP2* exhibit reduced expression in prostate cancer tissues [17, 18], whereas *ZIP6* and *ZIP10* are overexpressed in breast cancer tissues [19, 20]. *ZIP3* has also been linked to the onset of breast cancer [21]. Furthermore, in dermatological research, *ZIP2* has been associated with keratinocyte differentiation, suggesting its possibility as a therapeutic target for epidermal diseases [22, 23]. Notably, *ZIP12* has been identified to modulate hypoxia‐related pulmonary hypertension under hypoxic conditions and has been implicated in schizophrenia, with increased expression observed in the lateral prefrontal cortex [24]. Importantly, *ZIP8* has emerged as a critical hub gene in various studies, with previous research indicating its role in a multitude of physiological and pathological processes, including inflammatory responses and the degradation of the ECM [25, 26]. Building on these findings, our study seeks to investigate the mechanistic involvement of *ZIP* family genes, particularly *ZIP8*, with IDD by examining their mechanistic involvement.

The Wnt/β‐catenin signalling pathway is a crucial intracellular signalling network that plays a significant role in cell proliferation, differentiation and gene expression. In the context of IDD, the dysregulation of the Wnt/β‐catenin signalling pathway can lead to increased ECM degradation, thereby promoting the progression of IDD [27]. Furthermore, the Wnt/β‐catenin signalling pathway is closely associated with the apoptosis and proliferation of NP cells, and its dysregulation may lead to NP cell dysfunction, subsequently affecting the homeostasis of the intervertebral disc [28]. Therefore, the Wnt/β‐catenin signalling pathway is a promising target for therapeutic intervention in IDD.

Though knowledge of IDD has advanced, the exact molecular pathways are still unknown, especially about *ZIP8*'s involvement. Previous findings suggest that *ZIP8* influences inflammatory responses, ECM degradation and Wnt/β‐catenin signalling [29]. This research aims to examine the effects of *ZIP8* expression on IDD progression, focusing on its role in inflammation, ECM integrity and apoptosis in NP cells and animal models. Additionally, we explore the therapeutic potential of *ZIP8* knockdown and lithium chloride (LiCl) treatment in modulating these pathways. The aim was to elucidate the function of *ZIP8* in IDD and determine prospective therapy targets for degenerative disc disease.

## 2 Material and Methods

### 2.1 Analysis of IDD‐Related Differentially Expressed Genes (DEGs)

The GSE27494 dataset was downloaded from the Gene Expression Omnibus (GEO) database (http://www.ncbi.nlm.nih.gov/geo), built upon the GPL1352 framework, [U133_X3P] Affymetrix Human X3P Array. Among them, four disc cells in 3D control were regarded as the control group and four disc cells in 3D inflammatory cytokine interleukin‐1 (IL‐1) were regarded as the case group. Then, the GEO2R tool analysed the two groups of samples and set fold change (FC) greater than 2 as the screening condition for upregulation of DEGs and FC less than 0.5 as the screening condition for downregulation of DEGs, and both met *p* < 0.05.

### 2.2 Weighted Gene Co‐Expression Network Analysis (WGCNA) Analysis of the GSE27494 Dataset

ImageGP 2.0 database (https://www.bic.ac.cn/BIC/#/) was used to analyse all genes in the GSE27494 dataset, construct the network of gene co‐expression and determine the optimal soft‐threshold power to ensure that the network follows a scale‐free topology. Next, according to the adjacency function formed by the gene network, the contrast between module eigengenes (MEs) was computed, leading to the formation of a hierarchical clustering dendrogram. Then, according to the different expression levels of genes, they were divided into different gene modules. Finally, modules were assessed in relation to their association with the two sample groups within the GSE27494 dataset. The module that manifested the highest correlation coefficient was deemed the key module.

### 2.3 Screening and Enrichment Analysis of Common Genes

Bioinformatics (https://www.bioinformatics.com.cn/) database was used for cross‐analysis of the identified DEGs with genes in key modules to obtain a set of common genes. Subsequently, these common gene data were uploaded to the bioinformatics website (http://www.bioinformatics.com.cn/?keywords=GO) for enrichment analysis. This analysis encompassed pathways from the Kyoto Encyclopedia of Genes and Genomes (KEGG) and additionally, gene ontology (GO) terms, comprising biological processes (BPs), molecular functions (MFs) and cellular components (CCs). *p*‐values less than 0.05 were used to classify results as statistically significant.

### 2.4 Functional Analysis of the ZIP Family

We initially retrieved the *ZIP* family gene information from the GeneCards database (https://www.genecards.org/). After obtaining the genes, according to the protein sequences encoded by these genes, using the Search Tool for the Retrieval of Interacting Genes (STRING) database (https://string‐db.org/), we built a protein–protein interaction (PPI) network. Subsequently, the Gene Set Enrichment Analysis (GSEA) platform (http://www.gsea‐msigdb.org/gsea/downloads.jsp) was employed to conduct a WikiPathway enrichment analysis on the ZIP family genes. The purpose of this study was to identify possible biological pathways and functions connected to these genes. Only pathways and functions with *p* < 0.05 were deemed significant.

### 2.5 Analysis of ZIP8 Diagnosis and Pathway Significance in IDD

Utilising the Bioinformatics (https://www.bioinformatics.com.cn/) database, we investigated in depth the intersection between *ZIP8* family genes and a previously identified common gene, *SLC39A*8 (*ZIP8*). The expression of *ZIP8* in the GSE27494 dataset was subsequently examined. Thereafter, the ROC curve of *ZIP8* was analysed using the ‘tROC’ package. The area under the curve (AUC) was computed as an index to assess the clinical diagnostic relevance of genes. An AUC of more than 0.7 suggests that the gene performs well as a diagnostic in IDD. Additionally, to elucidate the potential biological pathways influenced by *ZIP8* expression, we performed GSEA for KEGG pathways, aiming to uncover the broader biological implications of *ZIP8* dysregulation in IDD.

### 2.6 Cell Sources

RAW 264.7 macrophages and human NP cells were from the National Collection of Authenticated Cell Cultures in China and ScienCell Research Laboratories, respectively. They were housed at 37°C and 5% CO_2_ in DMEM supplemented with 10% foetal bovine serum, 100 U/mL penicillin and 100 μg/mL streptomycin.

### 2.7 Cell Treatments

To simulate the inflammatory environment associated with IDD, human NP cells were given a 10‐ng/mL dose of interleukin 1β (IL‐1β) treatment to assess time‐dependent effects. In addition, NP cells were exposed to tumour necrosis factor‐alpha (TNF‐α) at a concentration of 20 ng/mL for 24 h to further mimic inflammatory conditions. For the investigation of Wnt/β‐catenin signalling, both NP cells and RAW 264.7 macrophages were handled with 10 mM LiCl for 24 h. XAV939 is a Wnt signalling pathway inhibitor. We selected a concentration of 10 μM for subsequent experiments and treated NP cells for 24 h. This treatment was designed to activate the Wnt/β‐catenin pathway and examine its function in the pathophysiology of IDD.

### 2.8 Preparation of Conditioned Medium (CM)

To induce M1 macrophage polarisation, RAW 264.7 macrophages were subjected to a 24‐h LPS treatment at a dose of 1 μg/mL. Following this induction, the supernatant was carefully removed, and to get rid of any remaining LPS, the cells were cleaned three times using phosphate‐buffered saline (PBS). The macrophages were then re‐cultured in a fresh DMEM complete medium for the next 24 h. To get rid of cell debris, the material was then collected and centrifuged at 1000× *g* for 10 min. The resulting superior, referred to as CM, was utilised in subsequent experiments to study its effects on NP cells.

### 2.9 Cell Transfection

For modulation of gene expression, lipofectamine 2000 was used to transfect cells, as per the manufacturer's recommendations. Specific small interfering RNAs (si‐RNAs) targeting *ZIP8* (si‐*ZIP8*‐1, si‐*ZIP8*‐2 and si‐*ZIP8*‐3) were presented to suppress its expression. Concurrently, a group under control was transfected with a non‐targeting si‐RNA (si‐NC) to serve as a negative control for the knockdown studies. An overexpression construct (over‐*ZIP8*) was utilised to enhance *ZIP8* expression in another subset of cells, with a vector control group established using an empty vector. After transfection, cells were maintained for an additional 48 h to achieve optimal gene expression changes before progressing to subsequent experimental evaluations.

### 2.10 Animal Model and IDD Induction

Sprague‐Dawley (SD) adult wild‐type rats were purchased from Shanghai SLAC Laboratory Animal Co. Ltd. All animal studies were carried out in compliance with the National Institutes of Health Guide for the Care and Use of Laboratory Animals and were authorised by the Animal Ethics Committee of Fudan University's Department of Laboratory Animal Science (Approval No. 2024‐MHYY‐55). The rats had full reign over food and water in a pathogen‐free environment with a 12‐h light–dark cycle. Thirty male rats, aged 12 weeks, were divided into three groups at random: normal (control), IDD and IDD + over‐*ZIP8* (*n* = 10 per group) (normal group: male SD rats that did not undergo any surgical procedures served as the normal control group, IDD group: rats in this group underwent the needle puncture technique to induce IDD but did not receive any adenoviral treatment and IDD + over‐ZIP8 group: rats in this group underwent both IDD induction and were treated with the Ad‐ZIP8 adenovirus to induce ZIP8 overexpression). To induce IDD, a well‐established needle puncture technique was employed. Rats were anaesthetised (40 mg/kg via intraperitoneal injection), and a dorsolateral approach was used to access and puncture the intervertebral discs, simulating IDD. A syringe needle (21G) was used to pierce the NP with the needle direction parallel to the cartilaginous endplate (CEP). The needle was rotated 360° in the NP and stayed for 1 min. For the *ZIP8* overexpression group, an adenovirus vector carrying the *ZIP8* gene (Ad‐*ZIP8*), purchased from FuNeng Gene Co. Ltd. in Guangzhou, was administered to induce gene overexpression in the affected discs. Microinjector syringes were used for adenovirus injection to reduce the injection volume error. The adenovirus titre was 1 × 10^8^ TU/mL. The rats were closely monitored postoperatively for recovery and general health status. After 12 weeks post induction, the animals were euthanised and the discs were harvested for subsequent analyses, including histological examination, molecular assays and imaging studies. The successful overexpression of ZIP8 was detected by assessing the expression levels of ZIP8 mRNA and protein in intervertebral disc tissues using qRT‐PCR and western blot (WB). The localisation and intensity of ZIP8 protein expression were further observed through IHC, which further validated our overexpression results.

### 2.11 Magnetic Resonance Imaging (MRI) Assessments

Twelve weeks post *ZIP8* overexpression induction, rats were subjected to MRI scans to evaluate the degree of disc degeneration. Animals were anaesthetised, and MRI scans were performed using a 7.0 Tesla scanner. The lumbar spine was imaged using T2‐weighted sagittal imaging. The degree of disc degeneration was evaluated based on signal intensity and disc height using standardised grading scales.

### 2.12 Enzyme‐Linked Immunosorbent Assay (ELISA)

Using specialised ELISA kits (Beyotime, Shanghai, China), the quantities of proinflammatory cytokines TNFα and IL‐6 in the supernatant were determined by the manufacturer's instructions. Using a microplate reader, the absorbance was measured at 450 nm. To account for optical flaws in the plate, the reference wavelength was set at 570 nm. Cytokine levels were determined by contrasting the sample absorbance with a reference curve derived from known concentrations of human IL‐6 and TNFα.

### 2.13 Quantitative Real‐Time Polymerase Chain Reaction (qRT‐PCR)

Following the manufacturer's instructions, total RNA was isolated from human NP cells, RAW 264.7 macrophages and rat intervertebral disc tissues using TRIzol reagent (Invitrogen, Shanghai, China). Using the SuperScript cDNA Synthesis Kit (ThermoFisher Scientific, Shanghai, China), the isolated RNA was reverse transcribed into complementary DNA (cDNA). qRT‐PCR amplification was conducted on a Bio‐Rad detection system utilising a SYBR Green dye‐based master mix. To accommodate species‐specific variations in gene sequences, different sets of primers were employed for each experimental context. Primers targeting human gene sequences were used for the in vitro (human NP cells) experiments, while primers for the in vivo (rat intervertebral disc tissues) and in vitro (RAW 264.7 macrophages) experiments were designed against rat and mouse gene sequences, respectively. These primer sequences are detailed in Table S1. We measured the levels of gene expression using the 2^−ΔΔCt^ technique, with *GAPDH* serving as the endogenous control to normalise the data. All qRT‐PCR assays were performed in triplicate to ensure data reliability and minimise bias.

### 2.14 WB Assay

Total protein was extracted from human NP cells, RAW 264.7 macrophages and rat intervertebral disc tissues using radioimmunoprecipitation assay lysis buffer. Using the BCA protein assay kit (Beyotime, Shanghai, China), we determined the protein content and used 1X loading buffer and DEPC water to adjust it to a concentration of 6 μg/μL.

Following electrophoresis on 10% SDS‐PAGE gels, the samples were transferred to polyvinylidene fluoride membranes. After being blocked for 1 h in PBST containing 5% non‐fat milk, the corresponding primary antibody was left on the membrane and incubated overnight at 4°C. The principal antibodies that were used were ZIP8 (1:1000), Bcl2 (1:500), Bax (1:750), Caspase3 (1:1000), p‐p65 (1:500), p65 (1:1000), p‐ERK (1:1000), COL II (1:1000), ggrecan (1:1000), ADAMTS5 (1:250), MMP13 (1:3000), β‐catenin (1:5000), IL‐10 (1:1000), COL2A1 (1:1000), ADAMTS5 (1:1000), A20 (1:1000, Abcam, China), IL‐1β (1:1000), COX‐2 (1:1000), iNOS (1:1000), IL‐6 (1:1000), TNF‐α (1:1000), IL‐4 (1:5000), C‐myc (1:5000), IκBα (1:5000), IKKβ (1:1000), p‐JNK (1:1000), p‐AKT (1:2000, Wuhan Sanying, China), p‐IκBα (1:1000, Affinity, China), p‐IKKα/β (1:1000, Affinity, China) and IKKα (1:1000, Affinity, China). The membranes were treated with the appropriate horseradish peroxidase (HRP)–conjugated secondary antibodies at a dilution of 1:5000 for 2 h at room temperature after the primary antibody incubation. The membranes were then thoroughly washed with PBST. A technique for enhanced chemiluminescence (ECL) detection was used to visualise the protein bands. Quantitative band intensities were analysed using ImageJ software (version 2.0.0), with protein levels normalised to GAPDH as loading controls.

### 2.15 Flow Cytometry

Apoptosis was assessed using the annexin V APC‐PI Apoptosis Detection Kit (KeyGen Biotech, Nanjing, China). After being collected, treated cells were again suspended in 500 μL of binding buffer. A total of 5 μL of annexin V‐fluorescein isothiocyanate (FITC) and 5 μL of propidium iodide (PI) were included in the suspension of cells. After mixing, it was incubated for 15 min in the dark to allow for adequate binding of the stains to apoptotic and necrotic cells. The percentage of apoptotic cells was then measured by flow cytometry analysis, and data analysis was done using the FlowJo program (version 10.0).

### 2.16 Cell Counting Kit 8 (CCK‐8) Assay

Using a CCK‐8, cell viability was evaluated in accordance with the manufacturer's recommendations. At specified time points (1, 2, 3, 4, and 5 days), the CCK‐8 solution was diluted and added to cells cultured in six‐well BioFlex plates. After incubating for 2 h at 37°C, the supernatant was moved to a 96‐well plate. Using an Infinite microplate reader (Infinite M200, Shanghai, China), the optical density (OD) of each well was measured at 450 nm.

### 2.17 Haematoxylin and Eosin (H&E) Staining

Tissue from the intervertebral disc was removed and preserved for a night in 10% formalin. Following sample embedding in paraffin, slices 5 μm thick were cut with a microtome. After that, sections were rehydrated using a graduated alcohol series after being deparaffinised in xylene. Haematoxylin and eosin staining was performed based on the customary procedure. After staining, sections were dehydrated, cleared in xylene and mounted for microscopic examination. Structural changes indicative of disc degeneration were analysed and scored based on established criteria.

### 2.18 Immunohistochemistry (IHC) Assay

For IHC staining, sections that had been deparaffinised and rehydrated were microwaved to retrieve antigens in citrate buffer (pH 6.0). After cooling, sections were blocked with 5% normal goat serum after being treated with 3% hydrogen peroxide to stop the endogenous peroxidase activity. Sections were then kept at 4°C for an entire night with primary antibodies against *ZIP8*, ADAMTS5, Aggrecan, MMP13, COL II and β‐catenin. After washing, secondary antibodies that were biotinylated were used, followed by incubation with the avidin–biotin complex. Diaminobenzidine (DAB) was used as a chromogen, resulting in brown‐coloured precipitates. After dehydrating, clearing and mounting the sections, they were counterstained with haematoxylin and examined under a light microscope.

### 2.19 TUNEL Staining

To assess apoptosis in the intervertebral disc tissues, TUNEL staining was done utilising a commercially available kit in compliance with the manufacturer's guidelines. In brief, deparaffinised and rehydrated sections were treated with proteinase K for antigen retrieval. Sections were then incubated with the TUNEL reaction mixture in a humidified chamber. After the incubation, converter‐peroxidase solution was added, followed by DAB substrate. Brown‐coloured cells indicated TUNEL‐positive apoptotic cells. Haematoxylin was used as a counterstain; sections were dried, cleaned in xylene and then mounted for microscopic inspection.

### 2.20 Statistical Analysis

Using R software, statistical analyses were carried out. Every assay's data were displayed as mean ± standard deviation. A post hoc test was used for multiple comparisons after the Student's *t*‐test or one‐way analysis of variance (ANOVA) was used to assess differences between the groups. Data from each assay were presented as the mean ± standard deviation (SD), and the standard error of the mean (SEM) was also calculated to reflect the precision of our estimates. *p* < 0.05 was deemed statistically significant in all analyses.

## 3 Results

### 3.1 The GSE27494 Dataset Samples Have the Strongest Connection With the Turquoise Module

The gene co‐expression network of the GSE27494 dataset was built using the R software's ‘WGCNA’ package, and the ideal soft threshold was found to be 18 (Figure S1A). Following this, we generated a trait heat map for the eight samples from the GSE27494 dataset (Figure S1B). Genes were categorised into distinct modules based on their expression variations, each represented by a specific colour. Both the cluster dendrogram and the eigengene adjacency heat map were constructed, as illustrated in Figure S1C,D. In the subsequent analysis of the GSE27494 dataset, the correlation between gene modules and their respective case and control groups was explored. Notably, MEturquoise demonstrated a strong positive correlation with the case samples (correlation coefficient of 0.98) and an equally strong negative correlation with the control samples (correlation coefficient of −0.98) (Figure S1E).

### 3.2 Enrichment Analysis of 186 Common Genes in the GSE27494 Dataset

**FIGURE 1:**
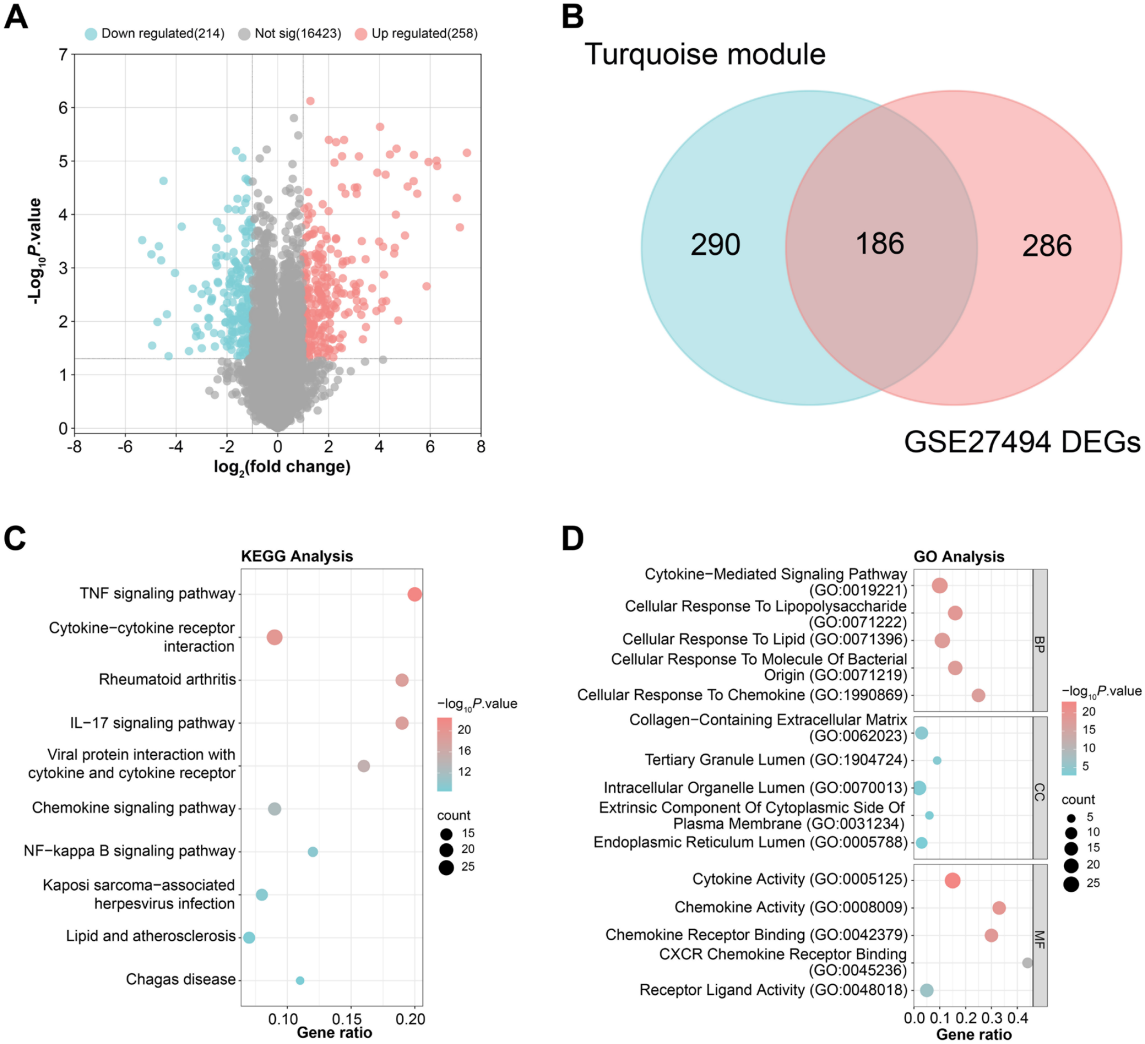
Enrichment analysis of 186 common genes associated with IDD. (A) Volcano map representation of DEGs in the GSE27494 dataset. The *x*‐axis represents the log_2_ fold change, while the y‐axis represents the negative base 10 logarithm of the adjusted *p*‐value. Upregulated DEGs in IDD are highlighted in red, downregulated DEGs in blue, and insignificant genes in gray. (B) Venn diagram depicting the overlap between genes identified in the turquoise module (476 genes on the left) and DEGs (472 genes on the right). (C and D) Bubble plots showing the enrichment analysis of 186 overlapping genes in KEGG and GO. The *y*‐axis represents specific terms within each category (KEGG, BP, CC, MF). The *x*‐axis usually represents significance (usually −log_10_ of the *p*‐value). The size of each bubble correlates with the number of enriched genes in each term, with larger bubbles representing higher enrichment and colour intensity indicating the significance level of the enrichment. IDD, intervertebral disc degeneration; DEGs, differentially expressed genes; KEGG, Kyoto Encyclopedia of Genes and Genomes; GO, gene ontology; BP, biological process; CC, cell component; MF, molecular function.

By the GEO2R tool, we identified 258 upregulated DEGs and 214 downregulated DEGs from the eight samples in the GSE27494 dataset, as illustrated in Figure 1A. Subsequently, out of these DEGs and the 476 genes from the MEturquoise, 186 common genes were selected (Figure 1B). Enrichment analysis indicated that the common genes were primarily connected to KEGG pathways such as the TNF signalling pathway, rheumatoid arthritis, cytokine–tokine receptor interaction, chemokine signalling pathway, IL‐17 signalling pathway and the NF‐kappa B signalling pathway (Figure 1C). Furthermore, according to GO enrichment analysis, these genes were more abundant in BP, CC and MF categories, including cytokine‐mediated signalling pathway, tertiary granule lumen, cellular response to chemokine, intracellular organelle lumen, cytokine activity and chemokine activity, among others (Figure 1D).

### 3.3 ZIP8 Overexpression in IDD and Its Implications for Clinical Diagnostics

**FIGURE 2:**
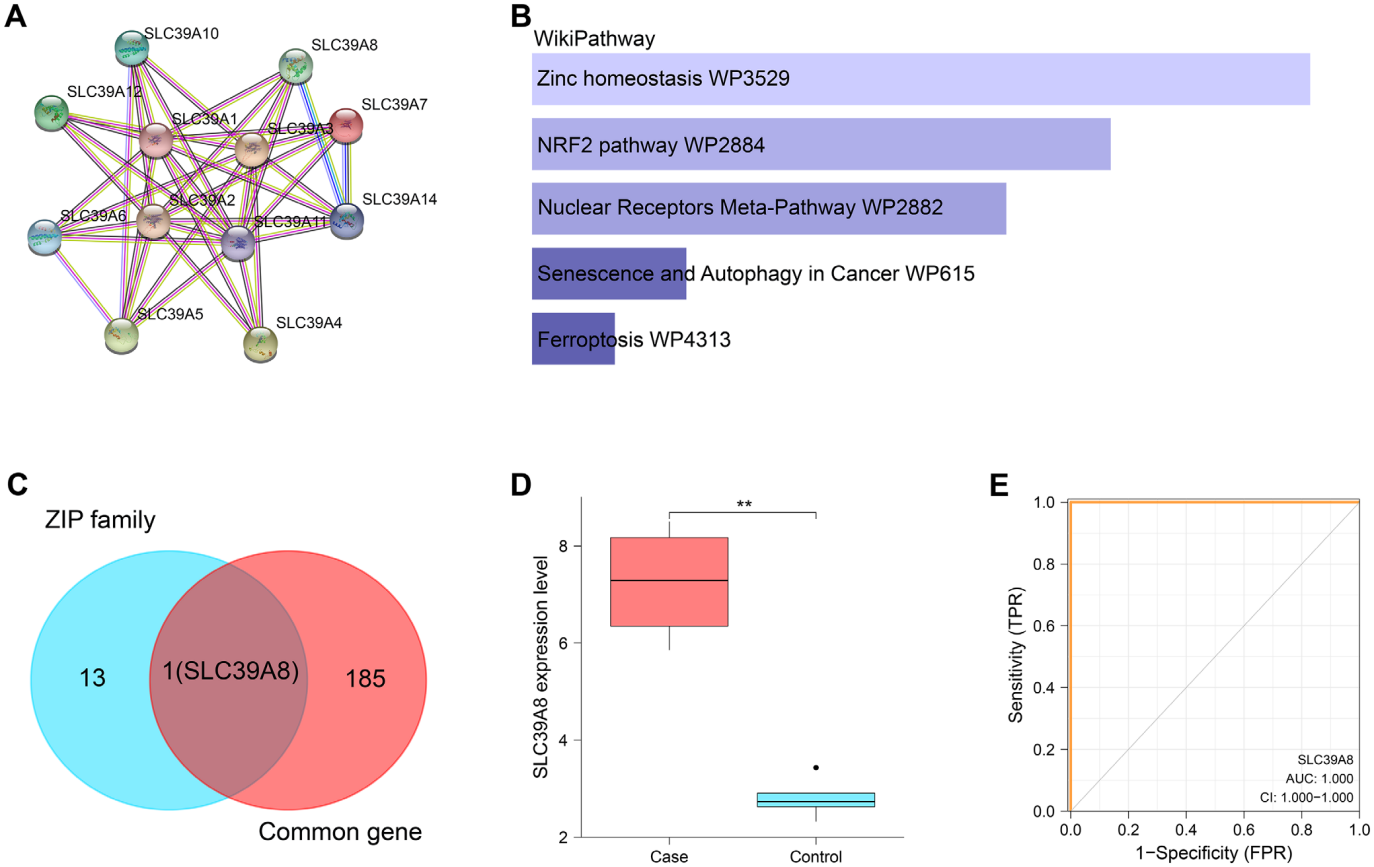
Analysis of the ZIP family genes. (A) PPI network of ZIP family genes. Each node (represented by a circle) represents an individual gene in the ZIP family, while connecting edges (lines) represent known or predicted interactions between these proteins. (B) GSEA–WikiPathway enrichment analysis of ZIP family genes. (C) Venn diagram, intersections (middle intersection) between ZIP family genes (left circle) and previously identified 186 common genes (right circle). (D) Boxplots representing the expression levels of SLC39A8 (ZIP8) in case and control samples from the GSE27494 dataset, with horizontal lines in each box representing the median. **p < 0.01. (E) ROC curve analysis of SLC39A8 (ZIP8) as a potential biomarker for IDD. The graph compares the true positive rate (sensitivity) to the false positive rate (1‐specificity) for different threshold levels. The AUC value close to 1 indicates the high diagnostic accuracy of ZIP8 in distinguishing IDD from healthy controls. PPI, protein–protein interaction; GSEA, Gene Set Enrichment Analysis; ROC, receiver operating characteristic; IDD, intervertebral disc degeneration; AUC, area under the curve.

The PPI network of Figure 2A indicated the interconnection among 12 ZIP family genes, including *SLC39A1*, *SLC39A2*, *SLC39A3*, *SLC39A4*, *SLC39A5*, *SLC39A6*, *SLC39A7*, *SLC39A8*, *SLC39A10*, *SLC39A11*, *SLC39A12* and *SLC39A14*. After WikiPathway enrichment analysis of these genes on the GSEA website, five pathways were obtained, namely zinc homeostasis (WP3529), senescence and autophagy in cancer (WP615), NRF2 pathway (WP2884), ferroptosis (WP4313) and nuclear receptors meta‐pathway (WP2882) (Figure 2B). From 186 common genes and the ZIP gene family, a key overlapping gene *SLC39A*8, also known as *ZIP8*, was identified (Figure 2C). Analysis revealed that this gene exhibited elevated expression in the GSE27494 case cohort, suggesting its potential role in IDD (Figure 2D). In addition, ROC curve analysis indicated that *ZIP8* has great clinical diagnostic potential in IDD, with an AUC value of 1.000 (Figure 2E).

### 3.4 ZIP8 Was Significantly Enriched in the WNT Signalling Pathway

After the GSEA–KEGG enrichment analysis of *ZIP8*, we obtained the results shown in Figure S2A–D. In these results, *ZIP8‐enriched* pathways included the T cell receptor signalling pathway, TGF beta signalling pathway, Toll‐like receptor signalling pathway, cell cycle, apoptosis, JAK STAT signalling pathway, ECM receptor interaction, pancreatic cancer, WNT signalling pathway, VEGF signalling pathway and so on. These findings elucidate the multifaceted involvement of *ZIP8* in cellular regulatory mechanisms and disease‐related pathways, emphasising its importance in diverse physiological as well as pathological settings.

### 3.5 ZIP8 Knockdown Reverses IL‐1β–Induced Proliferation, Apoptosis and Inflammatory Response in NP Cells

**FIGURE 3:**
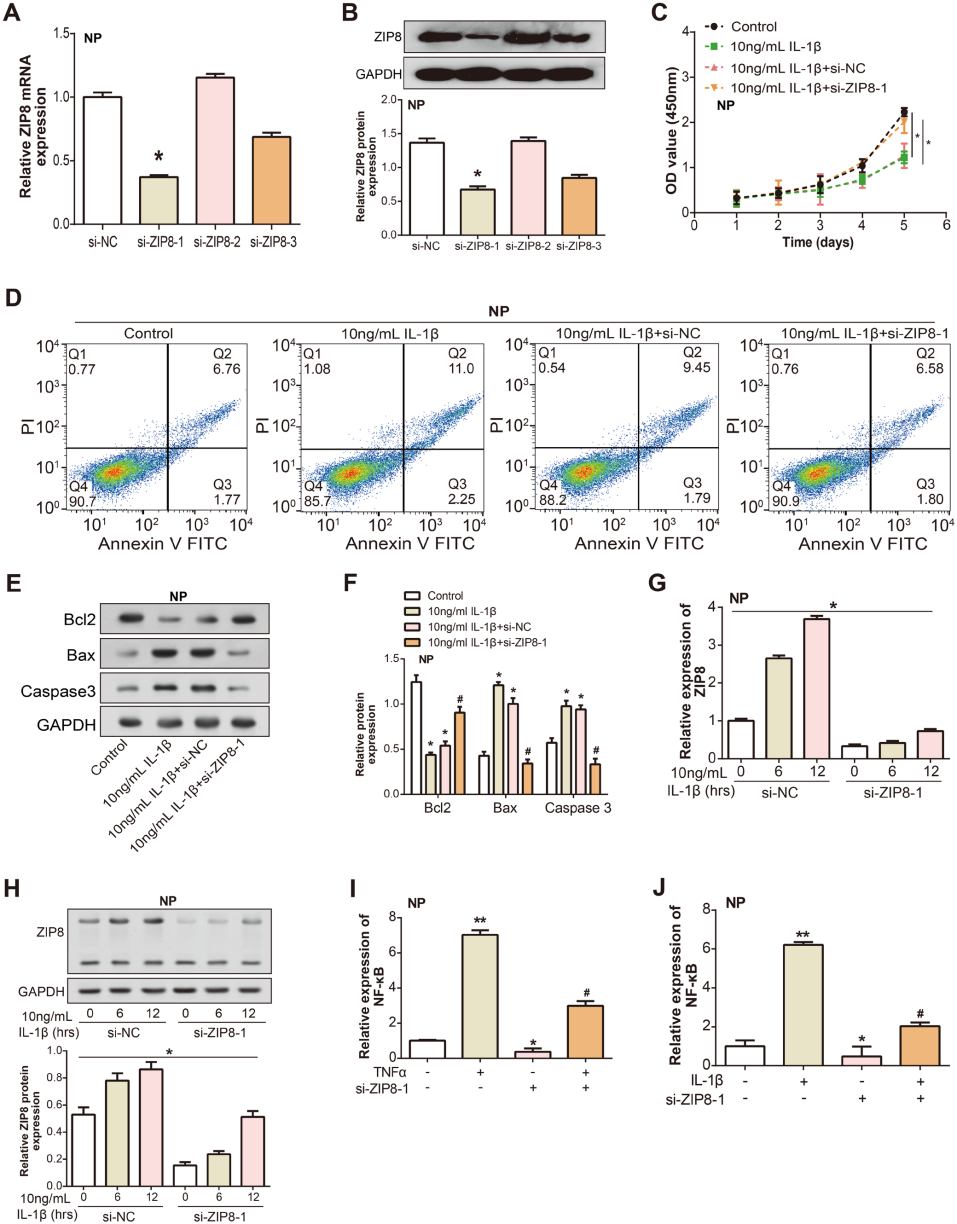
Effects of *ZIP8* knockdown on IL‐1β–induced alterations in NP cell proliferation, apoptosis and NF‐κB expression. (A) qRT‐PCR analysis of ZIP8 mRNA levels following treatment with three different ZIP8‐targeted si‐RNAs: si‐RNA1, si‐RNA2, and si‐RNA3. *p < 0.05. (B) WB analysis of ZIP8 protein levels in NP cells post‐treatment with si‐RNA1. The displayed bands correspond to ZIP8 and the loading control, underscoring the pronounced ZIP8 knockdown. *p < 0.05. (C) CCK‐8 assay proliferative response of NP cells after IL‐1β treatment in the presence of si‐ZIP8‐1 knockdown. Line segments of different colours represent different treatment groups, the *y*‐axis represents the OD value at 450 nm and the *x*‐axis represents different times in days. *p < 0.05. (D) Flow cytometric analysis of NP cells following IL‐1β treatment and concurrent ZIP8 silencing with si‐ZIP8‐1. The bottom left quadrant: viable cells (annexin V− and PI−), the top left quadrant: necrotic cells (annexin V− and PI+), the bottom right quadrant: early apoptotic cells (annexin V+ and PI−) and the top right quadrant: late apoptotic or secondary necrotic cells (annexin V+ and PI+). (E and F) WB analysis of apoptosis‐related proteins (Bcl2, Bax and Caspase3) in NP cells after IL‐1β treatment in the presence of si‐ZIP8‐1 knockdown. *p < 0.05 versus control, #*p* < 0.05 versus 10 ng/mL 1L‐1β + NC. (G and H) qRT‐PCR and WB detection of ZIP8 expression changes after 1L‐1β exposure for different periods (6 and 12 h) and after si‐ZIP8‐1 transfection. *p < 0.05. (I and J) qRT‐PCR analysis showed the expression levels of NF‐κB mRNA in NP cells treated with TNFα (D) or IL‐1β (E) with and without si‐ZIP8‐1 transfection. *p < 0.05 versus control, **p < 0.01 versus control, #*p* < 0.05 versus si‐ZIP8‐1. NP, nucleus pulposus; qRT‐PCR, quantitative real‐time polymerase chain reaction; WB, western blot; CCK‐8, cell counting kit‐8.

We evaluated the efficacy of three specific si‐RNAs aimed at *ZIP8*: si‐RNA1, si‐RNA2 and si‐RNA3. Among these, qRT‐PCR analyses clearly showed that si‐RNA1 provided the most pronounced knockdown effect on *ZIP8* expression (Figure 3A). This was further substantiated by WB, which unveiled a marked reduction in *ZIP8* protein levels post knockdown (Figure 3B). To recreate the inflammatory conditions characteristic of IDD, NP cells were treated with 10 ng/mL of IL‐1β. Subsequent cell viability assessments via the CCK‐8 assay revealed that IL‐1β significantly suppressed NP cell proliferation. However, intriguingly, the introduction of si‐*ZIP8*‐1 mitigated the way that IL‐1β inhibits cell growth (Figure 3C). Furthermore, flow cytometry analyses disclosed an enhanced apoptosis rate in NP cells post IL‐1β exposure. Yet, when *ZIP8* was concurrently silenced using si‐*ZIP8*‐1 in this IDD‐inspired setting, there was an observed reduction in the IL‐1β–induced apoptosis rate, reverting it closer to control levels (Figure 3D). A deeper dive into the protein landscape, facilitated by WB, indicated that IL‐1β modulated apoptotic markers by downregulating anti‐apoptotic Bcl2 while upregulating pro‐apoptotic factors Bax and Caspase3. Remarkably, these modulations were substantially counteracted upon co‐treatment with si‐*ZIP8*‐1 (Figure 3E,F).

*ZIP8* expression increased significantly after 6 and 12 h of IL‐1β exposure, and si‐*ZIP8*‐1–treated cells showed a decrease in *ZIP8* mRNA expression compared with si‐NC–treated controls (Figure 3G). WB analysis confirmed reduced protein levels of *ZIP8* after si‐RNA–mediated knockdown, which were in line with the gene expression results (Figure 3H). To assess the role of *ZIP8* in NF‐κB signalling, TNFα or IL‐1β was applied to NP cells, either with or without *ZIP8* knockdown. The results showed that TNFα significantly induced *NF‐κB* expression in NP cells. However, *ZIP8* knockdown via si‐*ZIP8*‐1 transfection substantially attenuated this induction (Figure 3I). Similarly, IL‐1β treatment elevated *NF‐κB* levels, which, when compared to the control (si‐NC) group, were considerably lower in the si‐*ZIP8*‐1 group (Figure 3J). These results imply that *ZIP8* is essential for modulating NF‐κB signalling in NP cells under inflammatory conditions.

### 3.6 ZIP8 Overexpression Regulates Inflammatory Responses and NF‐κB Signalling in NP Cells

**FIGURE 4:**
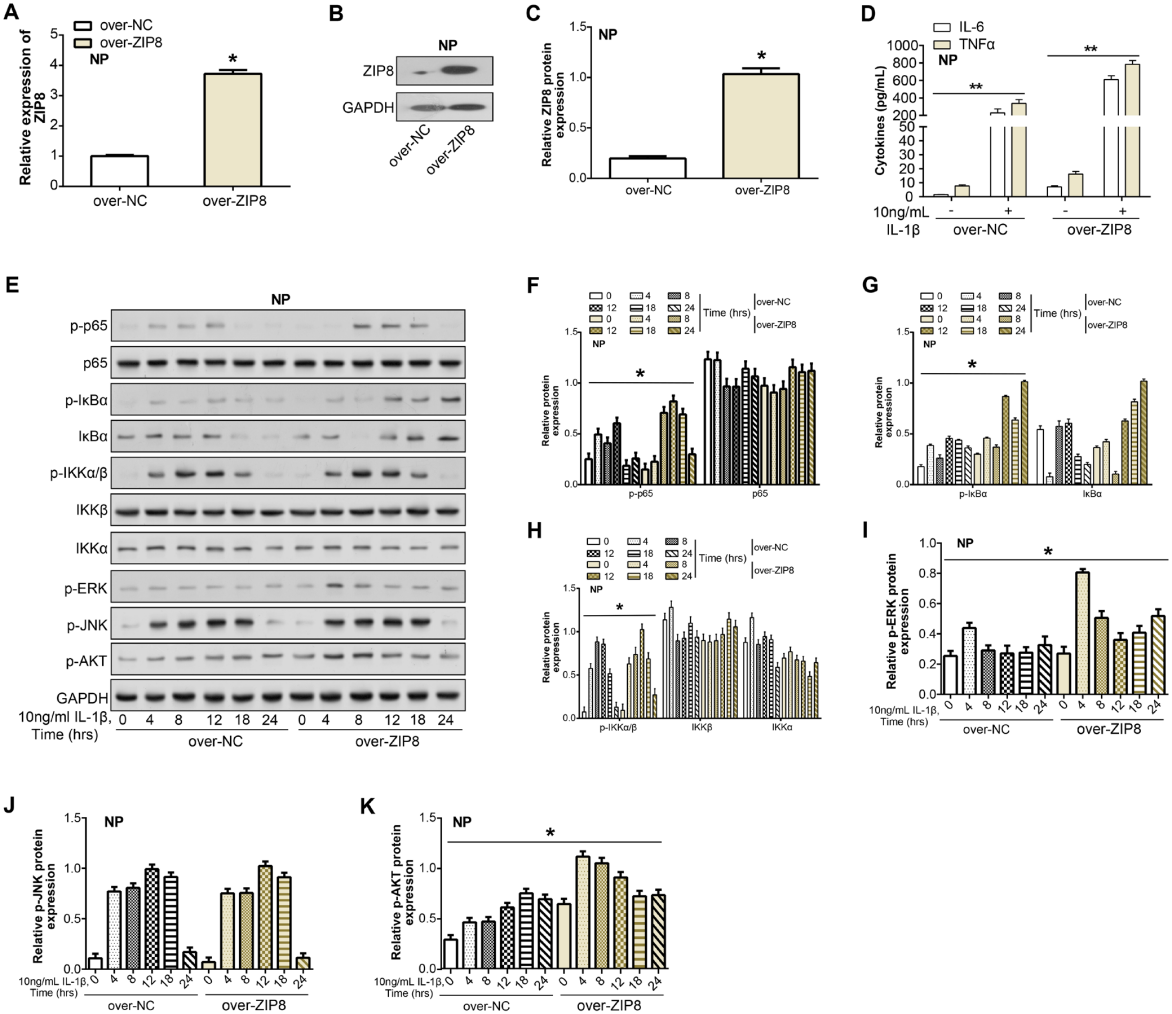
*ZIP8* overexpression regulates inflammatory responses and NF‐κB signalling in NP cells. (A–C) qRT‐PCR and WB detection of ZIP8 overexpression efficiency in NP cells. (D) ELISA assay to detect the expression of inflammatory factors (TNFα and IL‐6) in ZIP8 overexpressing cells in response to IL‐1β stimulation. (E–K) WB detection of changes in the expression of NF‐kB signalling pathway proteins after IL‐1β treatment for different periods and knockdown of ZIP8. *p < 0.05, **p < 0.01. NP, nucleus pulposus; qRT‐PCR, quantitative real‐time polymerase chain reaction; WB, western blot; ELISA, enzyme‐linked immunosorbent assay; hrs, hours.

qRT‐PCR and WB analyses confirmed the efficiency of *ZIP8* overexpression in NP cells (Figure 4A–C). When evaluating the cytokine response to IL‐1β stimulation, the ELISA findings revealed that the secretion of inflammatory factors (IL‐6, TGFα) in *ZIP8* overexpressing cells was much higher than that in the control group, suggesting that *ZIP8* plays a regulatory role in inflammation (Figure 4D). Further investigation into the NF‐κB signalling pathway revealed altered phosphorylation patterns of key signalling molecules in *ZIP8*‐overexpressing cells upon IL‐1β stimulation. Notably, the phosphorylation of p65, IκBα and IKKα/β was affected, indicating disruption of NF‐κB pathway activation (Figure 4E–K). In addition, the phosphorylation status of downstream signalling components (such as ERK and AKT) also changed, indicating that *ZIP8* is involved in the control of these pathways during the inflammatory reaction, but the phosphorylation level of JNK did not change significantly. Specifically, the reductions in p‐IκBα and p‐p65 suggest that *ZIP8* overexpression inhibits NF‐κB pathway activation.

### 3.7 ZIP8 Modulates IL‐1β–Induced Alterations in ECM Components and Wnt Signalling in NP Cells

**FIGURE 5:**
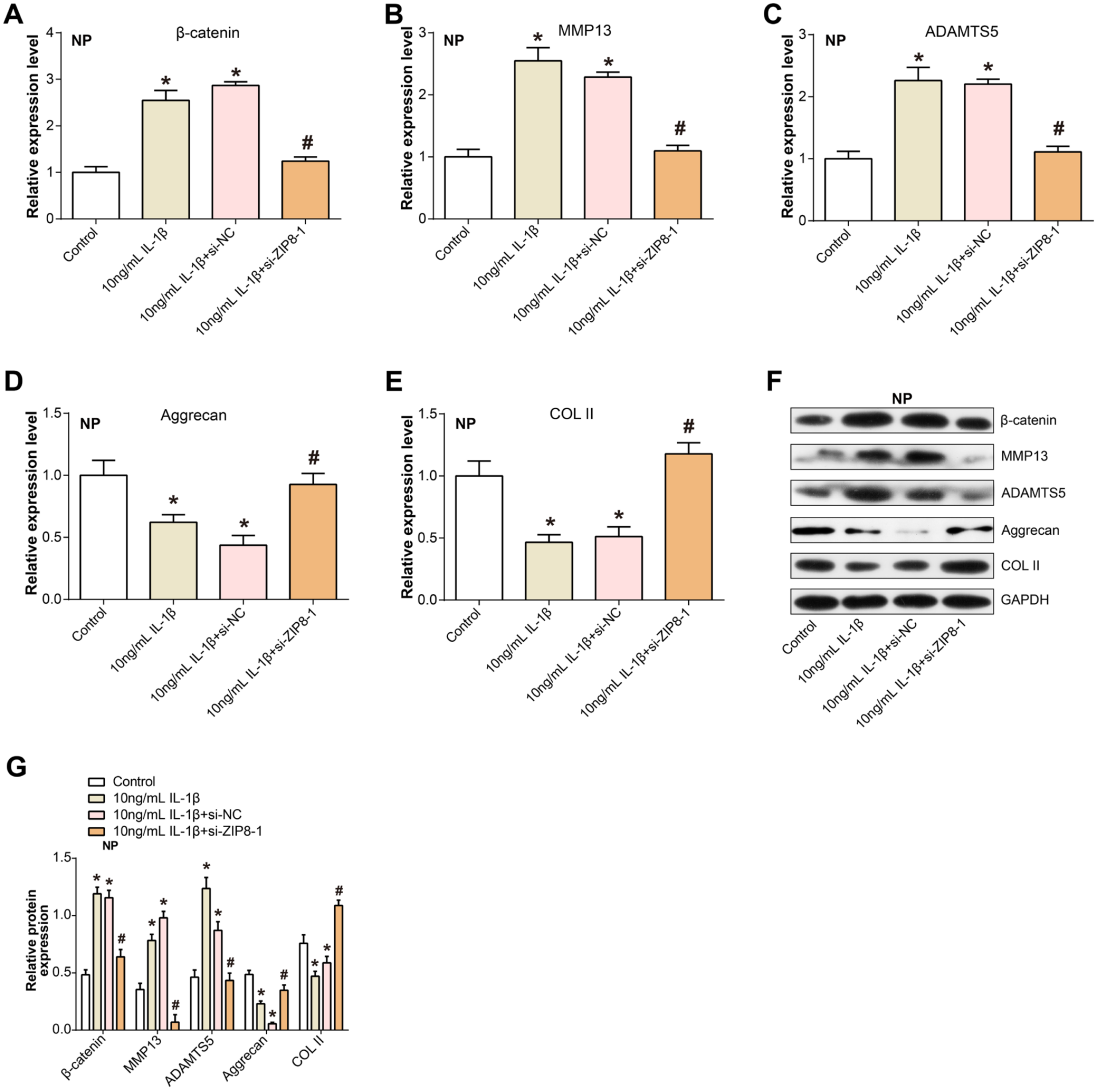
Effect of *ZIP8* on IL‐1β–driven changes in ECM composition and Wnt signalling in NP cells. (A–E) qRT‐PCR analysis of β‐catenin, MMP13, ADAMTS5, Aggrecan and COL II expression after IL‐1β exposure and ZIP8 co‐silencing. (F and G) WB analysis verifying differential protein expression of β‐catenin, MMP13, ADAMTS5, Aggrecan and COL II after IL‐1β exposure and ZIP8 co‐silencing. *p < 0.05 versus control, #*p* < 0.05 versus 10 ng/mL IL‐1β. NP, nucleus pulposus; qRT‐PCR, quantitative real‐time polymerase chain reaction; WB, western blot; ECM, extracellular matrix.

The integrity of the ECM within NP cells has a crucial function, and the control of its components is foundational [30]. To discern the impact of interleukin‐1β on key ECM constituents and the associated WNT signalling component, *β‐catenin*, we employed qRT‐PCR techniques. After IL‐1β treatment, we observed a notable upregulation of *β‐catenin*, a key player in the WNT signalling pathway. Concomitantly, there was a rise in the manifestation of genes involved in the degradation of the ECM, namely, *MMP13* and *ADAMTS5*. In contrast, the expression of ECM components, including *Aggrecan* and *COL II*, was markedly diminished. Intriguingly, co‐silencing of *ZIP8* significantly counteracted these IL‐1β–mediated effects (Figure 5A–E). To corroborate these findings and reinforce the robustness of our results, WB analyses were conducted (Figure 5F,G). Consistent with our qRT‐PCR results, protein evaluation reflected similar trends. These findings imply that *ZIP8* silencing possesses a shielding impact in maintaining ECM integrity.

### 3.8 ZIP8 Overexpression Exacerbates Disc Degeneration in the Rat Model

**FIGURE 6:**
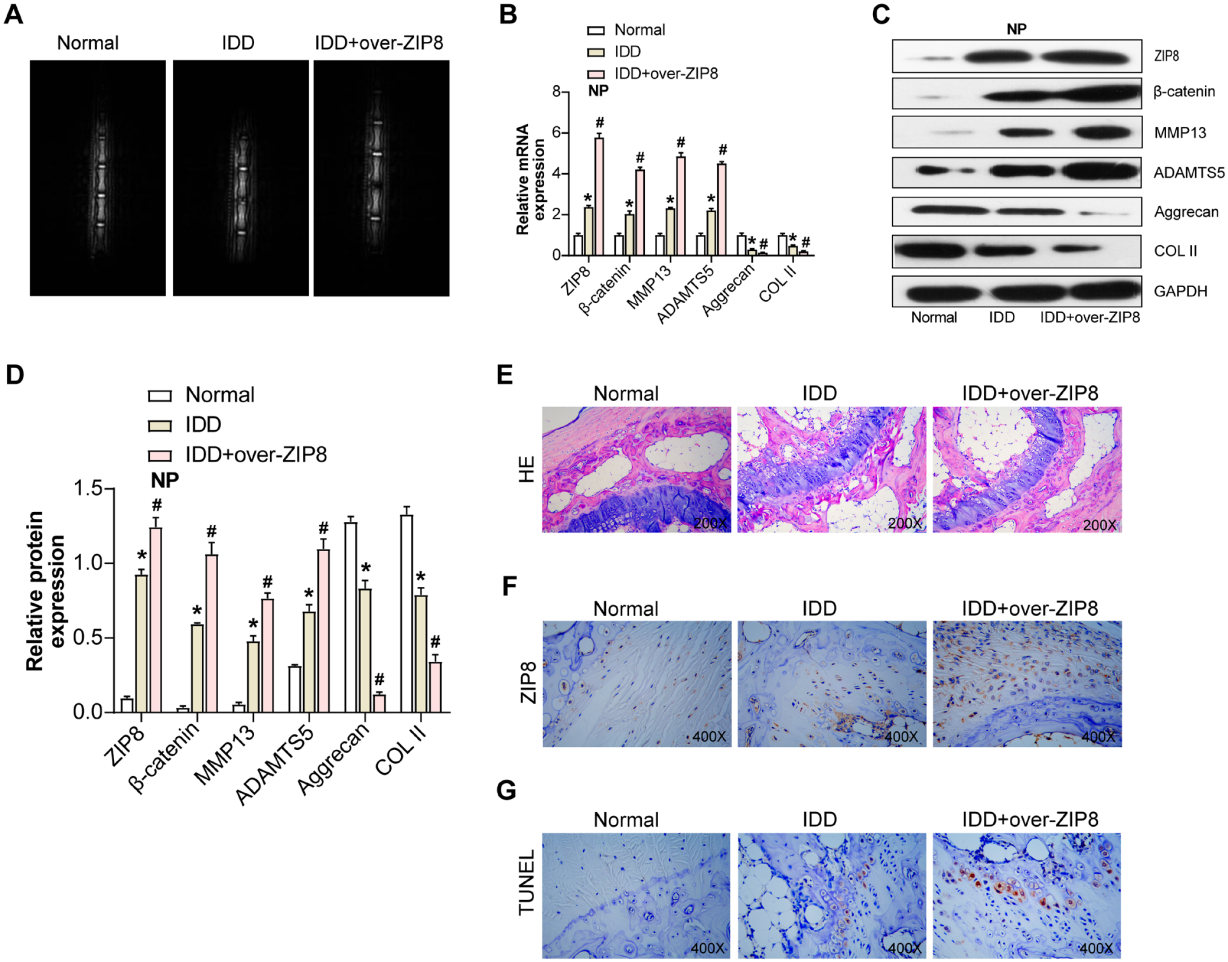
*ZIP8* overexpression exacerbates disc degeneration in SD rats. (A) MRI assessment of disc degeneration in wild‐type SD rats after ZIP8 overexpression. Representative images show obvious disc degeneration in the IDD + over‐ZIP8 group compared to the control IDD group. (B) qRT‐PCR analysis of the effect of ZIP8 overexpression on the expression of key matrix molecules and related proteins, including ZIP8, COL II, Aggrecan, ADAMTS5, MMP13 and β‐catenin. *p < 0.05. (C and D) Protein bands analysed by WB showed differential protein expression of ZIP8, COL II, Aggrecan, ADAMTS5, MMP13 and β‐catenin among the groups. *p < 0.05 versus control, #*p* < 0.05 versus IDD. (E) H&E staining of intervertebral disc tissues from normal, IDD and IDD + over‐ZIP8 groups. Areas with deep purple staining represent the nucleus pulposus, while those with lighter staining indicate the annulus fibrosus, with circles highlighting areas of notable degenerative changes. Magnification: 200X. (F) IHC analysis showcasing the expression pattern of ZIP8 across the normal, IDD and IDD + over‐ZIP8 groups. Dark brown staining indicates ZIP8‐positive regions, while lighter areas are ZIP8 negative, and circles emphasise regions of significantly elevated ZIP8 expression. Magnification: 400X. (G) TUNEL staining assessing cell apoptosis in the normal, IDD and IDD + over‐ZIP8 groups. Darkly stained spots represent TUNEL‐positive apoptotic cells, and circles underscore areas with a high concentration of apoptotic cells. Magnification: 400X. IDD, intervertebral disc degeneration; qRT‐PCR, quantitative real‐time polymerase chain reaction; WB, western blot; MRI, magnetic resonance imaging; SD, Sprague Dawley; H&E, haematoxylin and eosin; IHC, immunohistochemical.

Given the emergent roles of molecular modulators in vitro IDD dynamics, it was hypothesized that overexpression of *ZIP8* might exacerbate the progression of IDD in vivo. To rigorously test this proposition, we employed an IDD model using wild‐type SD rats. Twelve weeks post‐induction of *ZIP8* overexpression, MRI assessments delineated a more accentuated IDD in the IDD + over‐*ZIP8* group than in its control IDD counterpart, reinforcing the implication of *ZIP8* in IDD exacerbation (Figure 6A). Corroborating these imaging insights, qRT‐PCR analyses rendered a consistent molecular narrative. Overexpression of *ZIP8* was associated with a pronounced diminution in the expression of *COL II* and *Aggrecan* in the IDD model. Meanwhile, the significant upregulation of *ZIP8* expression was accompanied by an upregulation of *ADAMTS5*, *MMP13* and *β‐catenin* mRNA levels (Figure 6B). WB analyses mirrored these qRT‐PCR findings, further strengthening the molecular evidence and underscoring the congruence between transcriptional and translational alterations (Figure 6C,D).

H&E staining of the intervertebral disc tissues revealed substantial structural alterations. The architecture of the IDD group showed notable signs of disc degeneration compared to the normal group. Strikingly, this degeneration was even more pronounced in the IDD + over‐*ZIP8* group, indicating an exacerbated degenerative effect upon *ZIP8* overexpression (Figure 6E). IHC analysis of *ZIP8* expression highlighted distinct expression patterns across the groups. The normal tissue samples showed sporadic and faint *ZIP8* immunopositivity. In contrast, the IDD group evidenced enhanced *ZIP8* expression, a feature that was conspicuously amplified in the IDD + over‐*ZIP8* ensemble (Figure 6F). TUNEL staining was employed to gauge the cellular apoptotic events in the tissue samples. Cells from the normal group showed negligible apoptosis. However, the IDD group exhibited a marked increase in TUNEL‐positive cells, indicative of apoptosis. Intriguingly, the IDD + over‐*ZIP8* group displayed an even more intense apoptotic response, further underscoring the role of *ZIP8* in potentiating cell death in this context (Figure 6G).

### 3.9 ZIP8 Overexpression Enhances ECM Enzyme Activity in IDD

**FIGURE 7:**
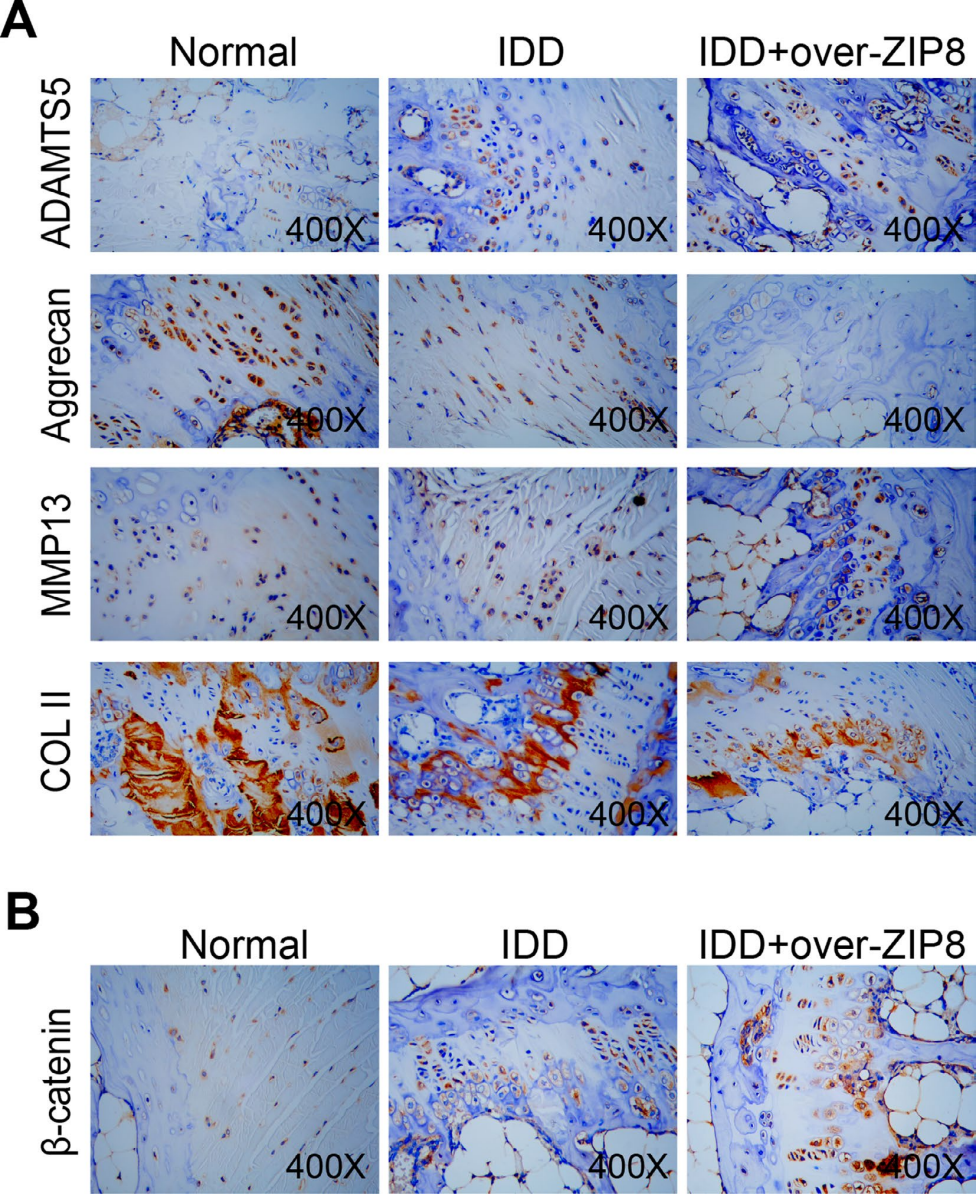
IHC staining demonstrates the effects of *ZIP8* overexpression on ECM proteins and β‐catenin activity in IDD. (A) Expression patterns of ADAMTS5, Aggrecan, MMP13 and Collagen II in the intervertebral disc tissues of the normal, IDD and IDD + over‐ZIP8 groups. Brown staining signifies the presence and intensity of these proteins, with denser staining indicating higher protein expression levels. Magnification: 400X. (B) Intervertebral disc tissues of the normal, IDD and IDD + over‐ZIP8 groups show staining for β‐catenin. The brown staining intensity correlates with the expression level of β‐catenin, with more pronounced staining suggesting elevated protein presence. Magnification: 400X. IDD, intervertebral disc degeneration; IHC, immunohistochemical.

By IHC staining, we looked at how key protein markers in the intervertebral disc tissues of the normal group, the IDD group and the IDD + over‐*ZIP8* group were expressed. Among them, ADAMTS5 was highest expressed in the *ZIP8*‐overexpressed IDD group, and this enhanced staining underscores the potential deleterious effect of elevated *ZIP8* levels on cartilage homeostasis. In contrast, aggrecan showed a very different pattern. Predominant brown staining was observed in the normal group, indicating that the intervertebral disc was in optimal health. However, there was a marked reduction in the IDD group and a further reduction in the presence of *ZIP8* overexpression. MMP13 showed an expression trend similar to ADAMTS5. The IDD of the *ZIP8* overexpression group showed the most extensive brown area, indicating enhanced matrix degradation mechanisms. COL II, the cornerstone of intervertebral disc structural stability, was found to be most abundantly expressed in the normal group. A decrease in its expression became apparent when progressing from IDD to IDD in a *ZIP8* overexpression state (Figure 7A). Of particular importance, β‐catenin emerged as an important marker. Its expression was raised in the IDD group concerning the normal group. This ascent was at its pinnacle in the *ZIP8*‐overexpressed IDD specimens, suggesting a possible interplay between *ZIP8* overexpression and IDD‐induced shifts in β‐catenin expression dynamics (Figure 7B).

### 3.10 ZIP8 Combined With XAV‐939 Affects IL‐1β–Induced NP Cell Proliferation and Apoptosis Through Wnt/β‐Catenin Signalling

**FIGURE 8:**
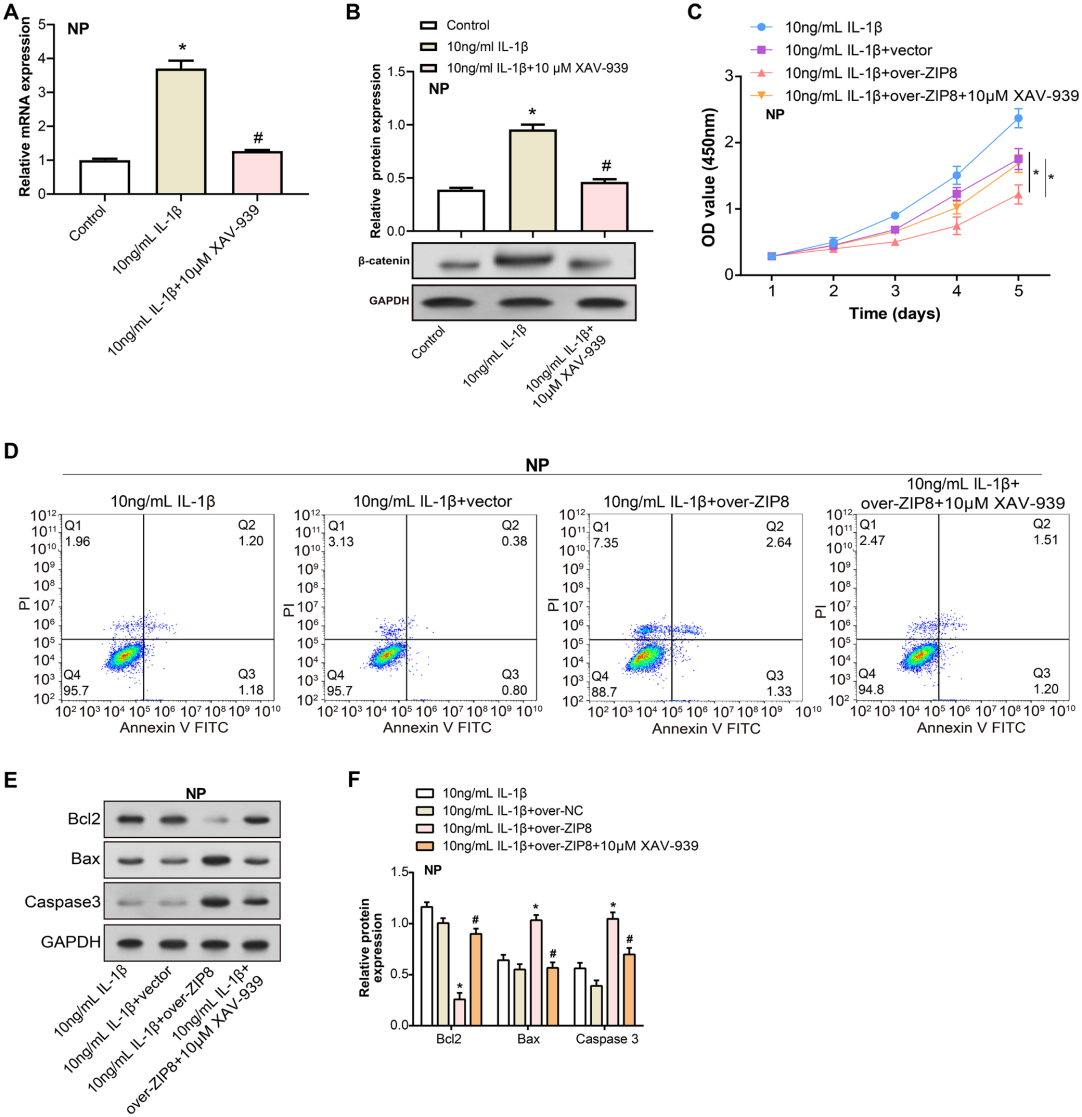
The interplay of *ZIP8* and XAV‐939 on IL‐1β–induced NP cell proliferation and apoptosis via Wnt/β‐catenin signalling. (A and B) qRT‐PCR and WB analyses of β‐catenin mRNA as well as protein levels in NP cells treated with IL‐1β (10 ng/mL) alone or in combination with XAV‐939 (10 mM).*p < 0.05 versus control, #*p* < 0.05 versus 10 ng/mL IL‐1β. (C) CCK‐8 assay assessing cell viability in NP cells treated with IL‐1β, IL‐1β + vector, IL‐1β + over‐ZIP8 and IL‐1β + over‐ZIP8 + XAV‐939 over 5 days. OD values were measured at 450 nm, indicating cell proliferation. *p < 0.05. (D) Flow cytometry plots showcasing the varying apoptosis rates across treatments: IL‐1β, IL‐1β with ZIP8 overexpression and the combined effect of IL‐1β, ZIP8 overexpression and XAV‐939. Differences in cell populations within distinct flow cytometry quadrants signify apoptotic shifts in response to treatments. (E and F) WB analyses of apoptosis‐associated proteins—Bax, Caspase3 and Bcl2, in cells subjected to IL‐1β, IL‐1β + ZIP8 overexpression and IL‐1β + ZIP8 overexpression + XAV‐939. Relative band intensities indicate modulation in protein expression levels across different experimental conditions. *p < 0.05 versus 10 ng/mL IL‐1β, #*p* < 0.05 verssu 10 ng/mL IL‐1β + over‐ZIP8. NP, nucleus pulposus; qRT‐PCR, quantitative real‐time polymerase chain reaction; WB, western blot; CCK‐8, cell counting kit‐8; OD, optical density.

Both qRT‐PCR and WB analyses consistently demonstrated that β‐catenin expression in NP cells exposed to IL‐1β treatment was notably diminished in contrast to the control. Notably, introducing the Wnt/β‐catenin pathway modulator XAV‐939 partially reduced the increased β‐catenin expression levels (Figure 8A,B). CCK‐8 assays showed that overexpression of ZIP8 further deepened the inhibition of cell proliferation caused by IL‐1β treatment alone. Interestingly, the addition of XAV‐939 partially alleviated this inhibition (Figure 8C). Flow cytometry evaluation showed a clear apoptotic gradient between the tested groups. Overexpression of ZIP8 further deepened the promotion of cell apoptosis caused by IL‐1β treatment alone, and the addition of XAV‐939 partially alleviated this promotion (Figure 8D). Subsequent examinations into apoptosis‐related protein expressions revealed that the IL‐1β + over‐*ZIP8* environment augmented Bax and Caspase3 levels while downregulating Bcl2. However, the incorporation of XAV‐939 appeared to counteract these modulations instigated by IL‐1β and overexpressed *ZIP8* (Figure 8E,F). In sum, our findings shed light on the intricate interplay between IL‐1β, *ZIP8* and XAV‐939 in modulating NP cell proliferation and apoptotic responses, suggesting potential therapeutic implications.

### 3.11 ZIP8 Knockdown Attenuated CM‐Induced Upregulation of Inflammatory Markers in RAW 264.7 Cells

**FIGURE 9:**
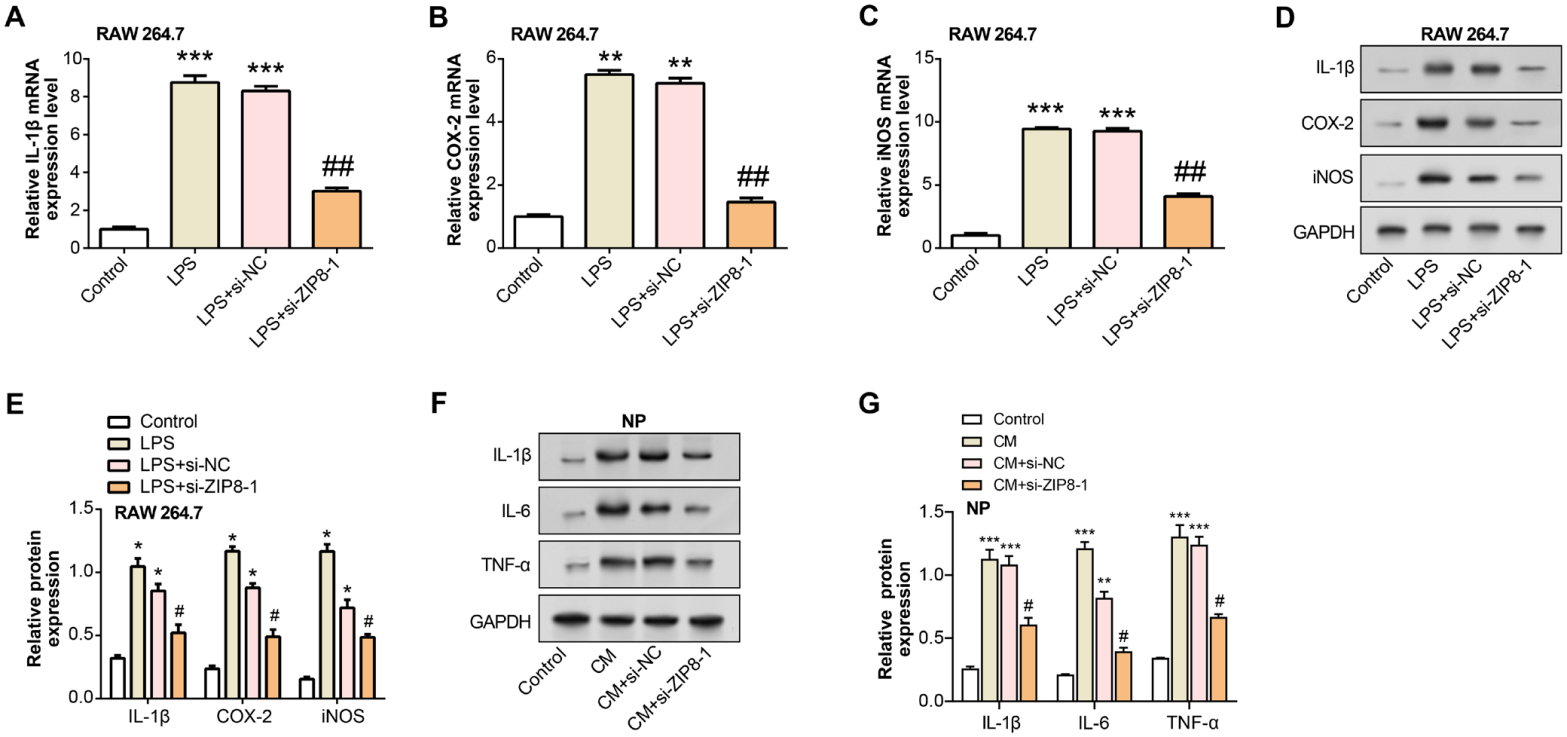
Knockdown of ZIP8 protects RAW 264.7 cells and NP cells from CM‐induced inflammation. (A–C) qRT‐PCR analysis of mRNA expression levels for IL‐1β (A), COX‐2 (B) and iNOS (C) in RAW 264.7 cells treated with LPS, with and without ZIP8 knockdown. **p < 0.01, ***p < 0.001 versus control, ##*p* < 0.01 versus LPS + si‐NC. (D and E) WB analysis showing protein levels of IL‐1β, COX‐2 and iNOS in the same treatment groups. *p < 0.05 versus control, #*p* < 0.05 versus LPS + si‐NC. (F and G) WB analysis showing the protein levels of IL‐1β, IL‐6 and TNF‐α in NP cells after CM treatment or knockdown of ZIP8. **p < 0.01 versus control group, ***p < 0.001 versus control group, #*p* < 0.05 versus CM + si‐NC group. qRT‐PCR, quantitative real‐time polymerase chain reaction; WB, western blot; LPS, lipopolysaccharide; C, conditioned medium; NP, nucleus pulposus.

The effects of *ZIP8* knockdown on inflammatory responses in RAW 264.7 macrophages were assessed by evaluating the expression levels of key inflammatory markers. qRT‐PCR revealed that LPS‐stimulated RAW 264.7 cells markedly elevated the mRNA levels of *iNOS*, *COX‐2* and *IL‐1β*. In contrast, transfection with si‐*ZIP8*‐1 effectively reduced the expression of these markers compared with both the control and LPS + si‐NC groups (Figure 9A–C). Consistently, WB analysis confirmed that the amounts of protein in IL‐1β, COX‐2 and iNOS were also elevated in response to LPS treatment but were significantly decreased following *ZIP8* knockdown (Figure 9D,E). In addition, we explored the effect of ZIP8 knockdown on NP cells exposed to CM of LPS‐treated RAW 264.7 macrophages. WB analysis showed that CM significantly increased the protein levels of proinflammatory cytokines IL‐1β, IL‐6 and TNF‐α in NP cells. ZIP8 knockdown in the CM + si‐ZIP8‐1 group led to a significant reduction in these inflammatory cytokines compared with the CM + si‐NC group (Figure 9F,G). These results indicate that ZIP8 knockdown alleviates LPS‐induced inflammatory responses in RAW 264.7 macrophages and reduces inflammatory cytokine expression in NP cells exposed to CM, highlighting the potential of ZIP8 as a therapeutic target for inflammation.

### 3.12 ZIP8 Silencing Inhibits M1 Macrophage Polarisation

The findings of qRT‐PCR indicated that the levels of TNF‐α and IL‐6 mRNA, markers of MI‐type macrophages, increased significantly after LPS stimulation, while *ZIP8* knockdown attenuated this increase. In contrast, anti‐inflammatory cytokines (*IL‐4*, *IL‐10*) were markedly elevated in the LPS + si‐*ZIP8*‐1 group in contrast to the control group and the LPS + si‐NC group, indicating an enhanced anti‐inflammatory response (Figure S3A–D). The degrees of protein expression of these cytokines were further confirmed by WB analysis. The findings demonstrated that the group treated with LPS had considerably higher levels of TNF‐α and IL‐6 protein, while *ZIP8* knockdown notably reduced these levels (Figure S3E,F). Conversely, the protein expression of IL‐4 and IL‐10 was significantly increased in the si‐*ZIP8*‐1 group. These results suggest that *ZIP8* knockdown modulates the cytokine profile in macrophages, lowering the expression of proinflammatory cytokines while raising the amounts of anti‐inflammatory cytokines. This modulation suggests that targeting *ZIP8* has a potential therapeutic role in inflammatory diseases.

### 3.13 ZIP8 Knockdown Alleviates ECM Degradation and Wnt/β‐Catenin Signalling in NP Cells Induced by CM Treatment

**FIGURE 10:**
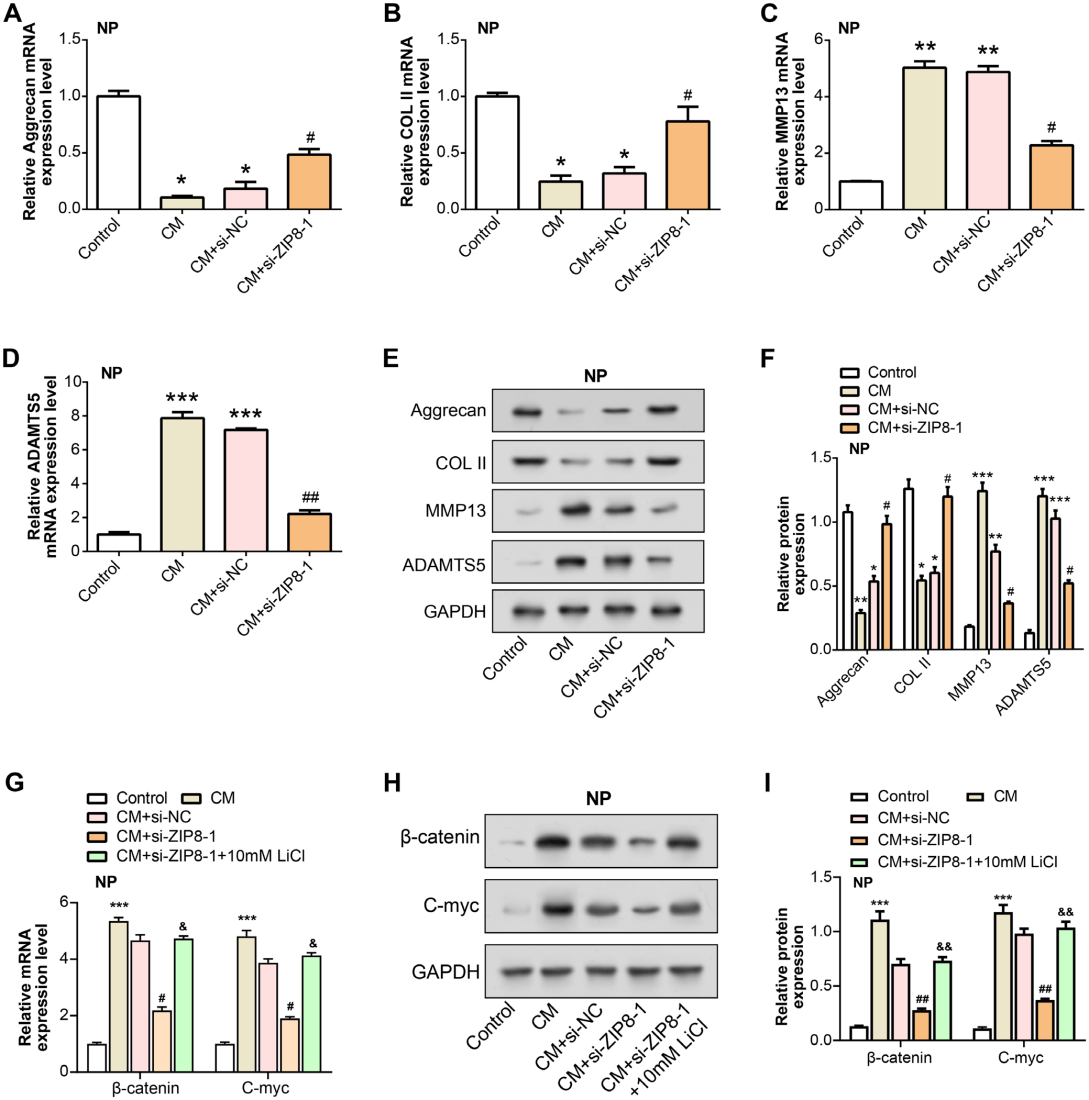
Knockdown of *ZIP8* alleviates ECM degradation and Wnt/β‐catenin signalling in NP cells induced by CM treatment. (A–D) qRT‐PCR results showing the relative mRNA expression levels of (A) Aggrecan, (B) COL II, (C) MMP13 and (D) ADAMTS5 in NP cells treated with control, CM, CM + si‐NC and CM + si‐ZIP8‐1. *p < 0.05 versus control group, **p < 0.01 versus control group, ***p < 0.001 versus control group, #*p* < 0.05 versus CM + si‐NC group, ##*p* < 0.01 versus CM + si‐NC group. (E and F) WB analysis shows the protein levels of Aggrecan, COL II, MMP13 and ADAMTS5 under the same treatment conditions. *p < 0.05 versus control group, **p < 0.01 versus control group, ***p < 0.001 versus control group, #*p* < 0.05 versus CM + si‐NC group. (G) Relative mRNA expression levels of β‐catenin and c‐myc in NP cells following CM treatment, ZIP8 knockdown (si‐ZIP8‐1) and LiCl treatment. ***p < 0.001 versus control group, #*p* < 0.05 versus CM + si‐NC group, &*p* < 0.05 versus CM + si‐ZIP8‐1 group. (H) WB analysis showing protein expression of β‐catenin and c‐myc in NP cells under the same conditions. (I) Quantification of the relative protein expression levels of β‐catenin and c‐myc in NP cells. ***p < 0.001 versus control group, ##*p* < 0.01 versus CM + si‐NC group, &&*p* < 0.01 versus CM + si‐ZIP8‐1 group. qRT‐PCR, quantitative real‐time polymerase chain reaction; WB, western blot; LPS, lipopolysaccharide; C, conditioned medium; NP, nucleus pulposus.

qRT‐PCR and WB analyses demonstrated that CM treatment led to a significant downregulation of ECM anabolic markers, including COL II and Aggrecan, and upregulation of ECM catabolic markers, such as MMP13 and ADAMTS5, in NP cells. Treatment with si‐*ZIP8‐1* mitigated these changes (Figure 10A–F). These data suggest that *ZIP8* knockdown counteracts CM‐induced cellular damage in NP cells by modulating the expression of markers associated with ECM synthesis and degradation, thereby protecting against M1 macrophage polarisation‐induced degradation.

Figure S4A demonstrated that LPS treatment dramatically enhanced mRNA expression levels of M1 macrophage markers (*IL‐6* and *TNF‐α*) in RAW 264.7 cells compared to the control group. When *ZIP8* expression was knocked down using si‐*ZIP8*‐1, the mRNA levels of *IL‐6* and *TNF‐α* were reduced considerably. When 10 mM LiCl was added, the inhibition of *IL‐6* and *TNF‐α* expression caused by ZIP8 knockdown was alleviated to a certain extent. In contrast, M2 macrophage markers (*IL‐4* and *IL‐10*) were downregulated after LPS treatment but significantly upregulated after *ZIP8* knockdown, and the expression of *IL‐4* and *IL‐10* was further decreased when LiCl was applied (Figure S4B). These results were also confirmed at the protein level (Figure S4C–E). Furthermore, *β‐catenin* and *C‐myc* levels in CM‐treated NP cells were considerably higher than those in negative control NP cells, as demonstrated by qRT‐PCR and WB. When si‐*ZIP8‐*1 therapy was added to NP cells treated with CM, the expression of *β‐catenin* and *C‐myc* was decreased. When comparing the CM + si‐*ZIP8*‐1 + LiCl–treated group to the CM + si‐*ZIP8*‐1 group, the levels of β‐catenin and C‐myc were offset by LiCl, an activator of the Wnt pathway (Figure 10F–H). These findings suggest that *ZIP8* knockdown regulates macrophage polarisation, and an important part of this process is the Wnt/β‐catenin pathway.

## 4 Discussion

IDD refers to the degeneration of the annulus fibrosus, NP and cartilage endplates of the intervertebral disc, resulting in changes such as atrophy, thinning, bulging as well as vertebral edge hyperplasia of the intervertebral disc [31]. Although patients may experience some improvement through massage, acupuncture, physical traction and closed acupuncture, existing therapeutic approaches are insufficient to cure IDD [32]. Our enrichment analysis of the 186 common genes identified from the GSE27494 dataset highlighted several key signalling pathways implicated in IDD, notably those related to inflammation. The strong association of these genes with the TNF and NF‐kappa B signalling pathways, crucial for inflammation and immune responses [33, 34], underscores the potential inflammatory underpinnings in IDD. This is consistent with previous findings, such as those by Liu et al., which demonstrated the anti‐inflammatory effects of fexofenadine in IDD [35]. ROC curve analysis highlighted the potential of *ZIP8* as a diagnostic marker for IDD, suggesting that further studies on *ZIP8* might result in fresh approaches to treatment and diagnosis.

The multifaceted roles of *ZIP8*, from zinc import during inflammation onset to its association with various congenital and musculoskeletal disorders, provide a compelling narrative of its centrality in cellular homeostasis. Interestingly, this gene's protein, which is glycosylated, mainly resides in the plasma membrane and mitochondria, reflecting its crucial roles in cellular dynamics [36, 37]. Beyond IDD, *ZIP8*'s implications in conditions like congenital disorders of glycosylation and hypermangnesemia with dystonia shed light on its extensive physiological importance [38, 39, 40]. Furthermore, its function in modulating pathogenic T‐cell responses during collagen‐induced rheumatoid arthritis and as a possible therapy objective for osteoarthritis accentuates its broader significance in musculoskeletal disorders [41]. Song et al. discovered that in OA pathogenesis, *ZIP8* is targeted by miR‐488 and is implicated in cartilage degradation [42]. Suppression of *ZIP8* in an OA animal model led to reduced cartilage degradation, further highlighting its significant role in OA progression. These results emphasise the broader impact of *ZIP8* on musculoskeletal diseases and its potential use as a target for therapy.

IL‐1β is a key cytokine produced by activated macrophages, which is essential to regulating immune responses and promoting other proinflammatory cytokines and enzymes, exacerbating inflammation [43]. In IDD, IL‐1β promotes ECM degradation and NP cell apoptosis, accelerating the degenerative process [44]. Studies have shown that IL‐1β induces NP cell apoptosis and inflammation, aggravating IDD. Zou et al. reported that *HO‐1* overexpression plays a protective role by blocking NF‐κB signalling to induce autophagy and inhibit IL‐1β–induced apoptosis [45]. Similarly, Zhu et al. demonstrated that higenamine alleviates IL‐1β–induced apoptosis by regulating the ROS‐mediated PI3K/Akt pathway [46]. Another study by Zhu et al. found that rhamnoside protected NPCs from IL‐1β–induced injury via lowering inflammation and apoptosis by ROS‐mediated inhibition of NF‐κB activation [47]. These findings suggest targeting these pathways as potential therapeutic approaches for IDD. Our study demonstrated that silencing *ZIP8* mitigated the inhibitory effects of IL‐1β on NP cell proliferation and apoptosis and counteracted the upregulation of NF‐κB induced by TNF‐α or IL‐1β. This suggests *ZIP8* as a potential therapeutic target for reducing apoptosis and inflammation in IDD. The potential of *ZIP8* as a therapeutic target for IDD is highlighted by its role in modulating inflammatory responses. Targeting *ZIP8* may offer a non‐surgical treatment option, utilising small molecule inhibitors or gene therapy to downregulate *ZIP8* activity, thereby reducing inflammation and maintaining the integrity of the intervertebral discs. This approach could alleviate pain and disability associated with IDD and ultimately improve patients' quality of life.

The ECM is a crucial network of proteins and glycosaminoglycans that provides structural support and regulates cell behaviour [48]. Key ECM components include aggrecan, a large proteoglycan that binds water to maintain disc hydration, and COL II, which provides tensile strength to the disc [49]. Enzymes such as MMP‐13 and ADAMTS5 are involved in ECM degradation; MMP‐13 degrades collagen fibres, while ADAMTS5 breaks down aggrecan [50]. In IDD, increased activity of these enzymes leads to excessive degradation of COL II and aggrecan, compromising ECM integrity. This imbalance contributes to disc dysfunction, decreased hydration and increased inflammation, exacerbating IDD progression. Zou et al. found that upregulated MMP activity disrupts ECM balance, leading to degeneration [51]. Similarly, Zhu et al. demonstrated that LINC00284 exacerbates IDD by enhancing ECM degradation through the miR‐205‐3p/Wnt/β‐catenin signalling axis, resulting in increased MMP activity and reduced ECM synthesis [52]. In our study, IL‐1β treatment significantly increased β‐catenin levels and the expression of ECM‐degrading enzymes MMP13 and ADAMTS5, while reducing ECM components Aggrecan and COL II. Knockdown of *ZIP8* countered these effects, indicating a protective role in maintaining ECM integrity. Additionally, *ZIP8* overexpression exacerbated IDD in rat models, with minimal *ZIP8* presence in normal tissues but significantly higher levels in the IDD group, correlating with increased degeneration severity. This was further supported by reduced *COL II* and *Aggrecan* levels and increased *ADAMTS5* and *MMP13* expression, highlighting the role of *ZIP8* in promoting ECM degradation.

The Wnt/β‐catenin signalling pathway plays a crucial role in regulating cell proliferation and differentiation [53]. In the context of IDD, Wnt signalling has been recognised as an essential modulator of disc cell function and matrix homeostasis. Dysregulation of Wnt signalling has been observed to result in increased catabolic activity, which in turn promotes ECM degradation and inflammation [54]. This results in the degeneration of intervertebral discs, which is characterised by a loss of disc height, reduced water content and compromised structural integrity. A deeper comprehension of, and ability to regulate, Wnt signalling may thus provide promising avenues for therapeutic intervention in the management of IDD. XAV‐939 is a potent inhibitor of Wnt/β‐catenin signalling that acts by stabilising the Axin–β‐catenin complex and promoting β‐catenin degradation, thereby reducing its accumulation and subsequent activation of target genes [55]. In the context of IDD, activation of Wnt/β‐catenin signalling has been associated with increased MMP expression and cartilage degradation. Our findings highlight the therapeutic potential of XAV‐939 in counteracting these effects. We observed that XAV‐939 effectively reduced IL‐1β–induced β‐catenin expression in NP cells, reduced apoptosis and mitigated the exacerbating effects of ZIP8 overexpression. These results suggest that inhibition of Wnt/β‐catenin signalling using XAV‐939 may provide a promising approach to prevent or alleviate IDD pathology. Additionally, the Wnt/β‐catenin pathway is connected with several other known IDD‐related pathways, forming a complex network of interactions that influence the pathogenesis of IDD. It interacts with pathways such as transforming growth factor‐beta (TGF‐β) and bone morphogenetic protein (BMP), affecting matrix synthesis and cell differentiation, and with NF‐κB, impacting cellular senescence and ECM metabolism in IDD [56, 57]. This crosstalk can regulate the expression of enzymes like MMPs and the production of inflammatory cytokines, thereby affecting disc health. Targeting these pathways may lead to therapies that modulate Wnt/β‐catenin signalling to improve the disc environment and potentially slow the progression of IDD. Future research should explore these interactions to develop a multi‐target IDD treatment strategy.

A CM is a medium containing cell‐secreted factors that reflect the physiological state of the cell [58]. In the context of IDD, CM from inflammatory cells can induce a proinflammatory environment in NP cells, mimicking IDD conditions [59]. Macrophage polarisation refers to their differentiation into different functional states: M1 (proinflammatory) and M2 (anti‐inflammatory). M1 macrophages release cytokines that exacerbate inflammation and tissue damage, being essential to the development of IDD [60]. Yang et al. demonstrated that different p38 MAPK isoforms in NP cells affect macrophage polarisation in IDD, with p38α and β promoting proinflammatory M1 macrophages, highlighting potential targets to reduce inflammation and slow down the progression of IDD [61]. In addition to the effects of inflammatory CM, our study also explored the impact of LiCl, a known activator of the Wnt/β‐catenin signalling pathway. LiCl activates this pathway by inhibiting glycogen synthase kinase‐3β (GSK‐3β), stabilising β‐catenin and promoting Wnt target gene activation [62]. This activation can worsen cartilage damage by increasing MMP expression and disrupting the balance between cartilage synthesis and degradation [63]. Yu et al. found that LiCl impairs chondrogenesis and cartilage regeneration in osteoarthritis (OA) models through enhanced β‐catenin activity, whereas strontium ranelate promotes cartilage repair by inhibiting this pathway [64]. LiCl also affects Hedgehog signalling by elongating chondrocyte primary cilia and modulates inflammatory responses in articular chondrocytes by reducing IL‐1β– and TNF‐α–induced increases in MMP‐13 and PGE2 [65]. Our study demonstrated that knockdown of ZIP8 attenuated the upregulation of inflammatory markers induced by LPS in RAW 264.7 cells. Inhibition of ZIP8 suppressed M1 polarisation in macrophages and alleviated ECM degradation in CM‐treated NP cells. In addition, *ZIP8* knockdown and Wnt pathway inhibition regulated macrophage polarisation and β‐catenin signalling in RAW 264.7 cells. This suggests that the interaction between CM‐induced macrophage polarisation and ECM degradation is critical for understanding and potentially alleviating IDD pathology.

In this study, we recognise several limitations that warrant consideration. Our use of a rat model to investigate IDD may not encapsulate the full spectrum of the human condition due to species‐specific differences in anatomy, physiology and disease progression, potentially affecting the generalisability of our findings. Additionally, our focus on *ZIP8* overexpression rather than knockdown in the in vivo IDD model provides insights into its role in exacerbating disc degeneration but does not directly address the therapeutic potential of *ZIP8* inhibition. Future studies will concentrate on *ZIP8* knockdown to ascertain its protective effects in IDD, which is crucial for establishing *ZIP8* as a viable therapeutic target. We will explore whether reducing ZIP8 expression can alleviate inflammation, preserve ECM integrity and mitigate disc degeneration, thus providing a more robust foundation for targeted therapies. Furthermore, the potential off‐target effects and compensatory mechanisms associated with *ZIP8* modulation require further investigation. These may involve unintended impacts on gene regulation networks, signal transduction pathways and cellular functions, as well as the upregulation of other zinc transporters, readjustments in cellular signalling and ECM remodelling. Understanding these complexities is vital for the development of targeted IDD therapies. Looking ahead, future research directions will include translating *ZIP8* inhibition into targeted therapies, with a focus on human‐specific dosing, safety assessments and efficacy trials. We will also investigate the long‐term effects and potential side effects of *ZIP8* modulation in clinical settings. Additionally, exploring the role of *ZIP8* in other diseases or degenerative conditions could unveil broader therapeutic applications. We advocate for studies utilising human cell cultures, larger animal models and well‐designed clinical trials to validate the therapeutic potential of *ZIP8*‐targeted interventions in IDD and beyond.

## 5 Conclusion

**FIGURE 11:**
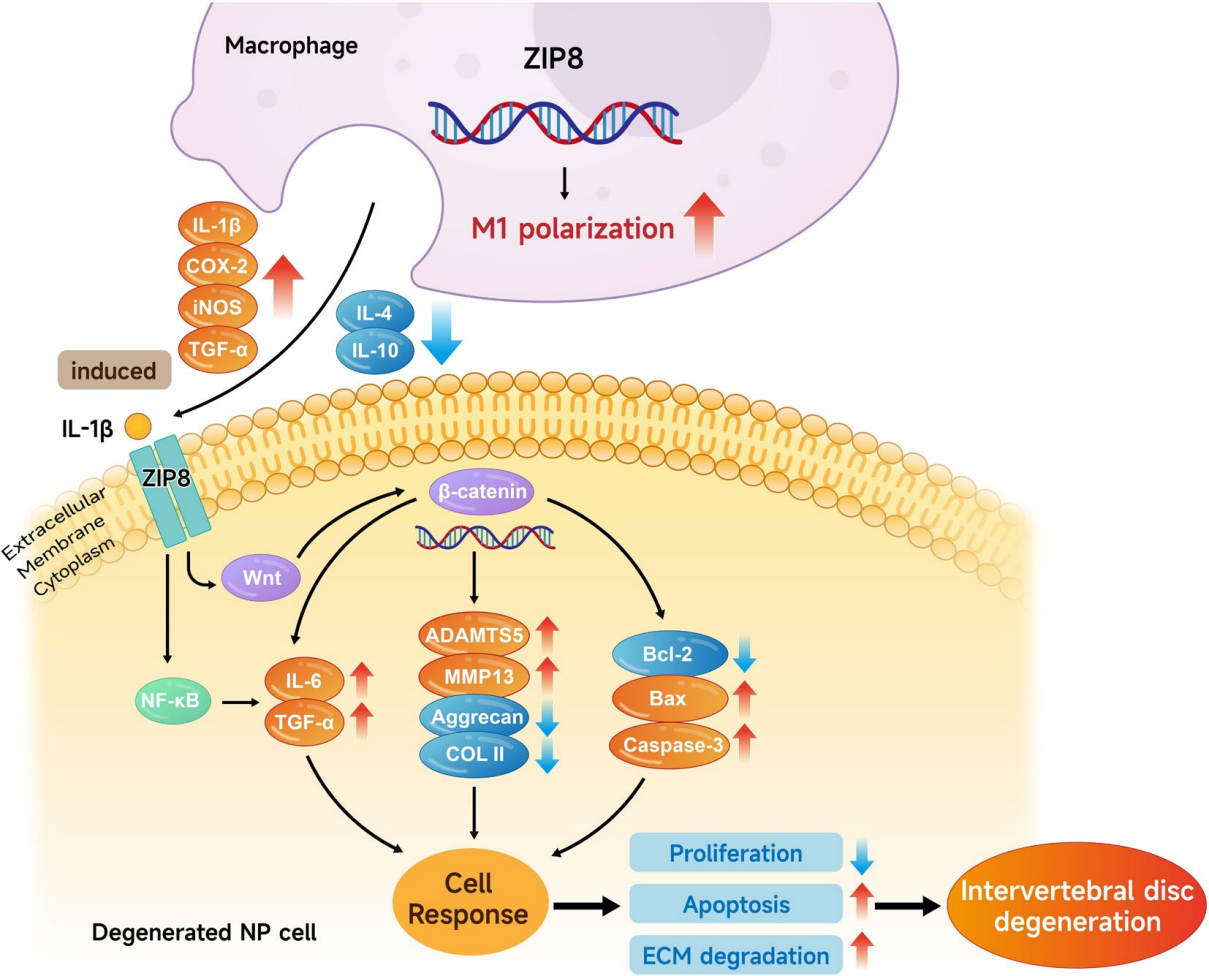
Mechanism of action of ZIP8 in macrophage polarisation and IDD. ZIP8 promotes M1 macrophage polarisation, leading to increased production of proinflammatory cytokines like IL‐1β, COX‐2 and iNOS. These cytokines induce IL‐1β in NP (nucleus pulposus) cells, which activates NF‐κB and Wnt/β‐catenin pathways. This results in increased expression of inflammatory mediators (IL‐6, TGF‐α), ECM‐degrading enzymes (ADAMTS5, MMP13) and apoptosis‐related proteins (Bax, caspase‐3), while reducing ECM components (aggrecan, COL II) and proliferation. These changes contribute to cell apoptosis, ECM degradation and IDD progression. IDD, intervertebral disc degeneration; ECM, extracellular matrix; NP, nucleus pulposus.

The importance of *ZIP8* in the pathophysiology of IDD is shown by this work. Our findings demonstrate that *ZIP8* overexpression exacerbates IDD by enhancing inflammatory responses, promoting ECM degradation and increasing apoptosis in NP cells (Figure 11). Conversely, *ZIP8* knockdown mitigates these effects, suggesting its potential as a therapeutic target for IDD. Specifically, *ZIP8* modulates key pathways, including the Wnt/β‐catenin and NF‐κB signalling pathways, impacting cell proliferation, apoptosis and cytokine production. The combined use of *ZIP8* knockdown and LiCl treatment further highlights the therapeutic potential by reducing inflammatory markers and modulating macrophage polarisation. These insights underscore the significance of targeting *ZIP8* in developing strategies to prevent or treat IDD, offering a promising avenue for future therapeutic interventions. *ZIP8*‐targeted therapies may offer a novel, personalised approach to IDD treatment by modulating inflammation and preserving ECM, potentially enhancing patient outcomes and slowing disease progression.

## Author Contributions

**Jun Xu:** conceptualization (equal), formal analysis (equal), writing – original draft (equal), writing – review and editing (equal). **Huijie Gu:** conceptualization (equal), data curation (equal), funding acquisition (equal), writing – original draft (equal), writing – review and editing (equal). **Kaifeng Zhou:** conceptualization (equal), writing – original draft (equal), writing – review and editing (equal). **Liang Wu:** conceptualization (equal), formal analysis (equal), funding acquisition (equal), investigation (equal), writing – original draft (equal). **Yiming Zhang:** conceptualization (equal), formal analysis (equal), supervision (equal), writing – original draft (equal). **Chong Bian:** data curation (equal), formal analysis (equal), funding acquisition (equal), writing – original draft (equal). **Zhongyue Huang:** formal analysis (equal), funding acquisition (equal), supervision (equal), writing – original draft (equal). **Guangnan Chen:** conceptualization (equal), formal analysis (equal), software (equal), writing – review and editing (equal). **Xiangyang Cheng:** conceptualization (equal), data curation (equal), methodology (equal), supervision (equal), writing – original draft (equal), writing – review and editing (equal). **Xiaofan Yin:** conceptualization (equal), data curation (equal), validation (equal), writing – review and editing (equal).

## Ethics Statement

The Animal Ethics Committee of the Department of Laboratory Animal Science, Fudan University, approved all animal experiments (2024‐MHYY‐55). They were conducted according to the guidelines set forth by the National Institutes of Health Guide for the Care and Use of Laboratory Animals.

## Conflicts of Interest

The authors declare no conflicts of interest.

## Supporting information


**Figure S1.** WGCNA analysis of the GSE2749 dataset. (A) Soft‐threshold power analysis of scale‐free topological model fit indices and mean connectivity. (B) Dendrogram of hierarchical clustering of samples, with each branch representing an individual sample. (C) Hierarchical clustering dendrogram of genes, displaying distinct colour‐coded modules that represent clusters of co‐expressed genes. Each branch corresponds to a gene, and the colour‐coded modules below signify groups of genes with similar expression patterns. (D) Module–trait relationships visualised as a dendrogram. The top dendrogram shows the relationship between modules and operating systems, and the bottom heat map shows the feature adjacency of gene modules. (E) Heat map of associations between gene modules and GSE27494 samples. Each row represents a gene module, while columns correspond to samples, and the colour intensity indicates the strength of the association. WGCNA, weighted gene co‐expression network analysis.

**Figure S2.** GSEA–KEGG enrichment analysis of *ZIP8*. (A–D) The top of the picture is the line graph of the enrichment score of *ZIP8*, the middle is the gene position map and the bottom is the change in the gene before and after treatment. GSEA, Gene Set Enrichment Analysis; KEGG, Kyoto Encyclopedia of Genes and Genomes.

**Figure S3.** Knockdown of *ZIP8* inhibits M1 polarisation. (A–D) Relative mRNA expression levels of proinflammatory cytokines *IL‐6* (A), *TNF‐α* (B) and anti‐inflammatory cytokines *IL‐4* (C) and *IL‐10* (D) in RAW 264.7 cells treated with LPS, LPS + si‐NC or LPS + si‐ZIP8‐1, as measured by qRT‐PCR. *p < 0.05, ***p < 0.001 versus control, ^#^ *p* < 0.05 versus LPS + si‐NC group. (E and F) WB analysis of IL‐6, TNF‐α, IL‐4 and IL‐10 protein levels in RAW 264.7 cells under the same conditions. **p < 0.01 versus control group, ***p < 0.001 versus control group, #*p* < 0.05, ##*p* < 0.01 versus LPS + si‐NC group. qRT‐PCR, quantitative real‐time polymerase chain reaction; WB, western blot; LPS, lipopolysaccharide.

**Figure S4.** Effects of *ZIP8* knockdown and LiCl treatment on cytokine expression in RAW 264.7 cells. (A and B) Relative mRNA expression levels of M1 macrophage markers (*IL‐6* and *TNF‐α*) (A) and M2 macrophage markers (*IL‐4* and *IL‐10*) (B) in RAW 264.7 cells under LPS stimulation, *ZIP8* knockdown (si‐*ZIP8*‐1) and LiCl treatment. **p < 0.01 versus control group, ***p < 0.001 versus control group, ^#^ *p* < 0.05 versus LPS + si‐NC group, ^##^ *p* < 0.01 versus LPS + si‐NC group, ^&^ *p* < 0.05 vs. LPS + si‐*ZIP8*‐1group, ^&&^ *p* < 0.01 vs. LPS + si‐*ZIP8*‐1 group. (C) WB analysis of IL‐6, TNF‐α, IL‐4 and IL‐10 in RAW 264.7 cells across the same conditions. (D and E) Quantification of the relative protein expression levels of M1 macrophage markers (IL‐6, TNF‐α) (D) and M2 macrophage markers (IL‐4, IL‐10) (E) in RAW 264.7 cells. ***p < 0.001 versus control group, ^#^ *p* < 0.05 versus LPS + si‐NC group, ^##^ *p* < 0.01 versus LPS + si‐NC group, ^&^ *p* < 0.05 versus LPS + si‐*ZIP8*‐1 group, ^&&^ *p* < 0.01 versus LPS + si‐*ZIP8*‐1 group, ^&&&^ *p* < 0.001 versus LPS + si‐*ZIP8*‐1 group. qRT‐PCR, quantitative real‐time polymerase chain reaction; WB, western blot; LPS, lipopolysaccharide.

**Table S1.** Human NP (nucleus pulposus) cells’ primer sequences.

**Table S2.** Rat intervertebral disc tissue primer sequences.

**Table S3.** Mouse RAW 264.7 macrophages’ primer sequences.

## Data Availability

The datasets used and/or analysed during the current study are available from the corresponding author upon reasonable request.
