# Supplementary figures and images for: ZIP8 Regulates Inflammation and Macrophage Polarisation in Intervertebral Disc Degeneration via the Wnt/β‐Catenin Pathway
Source: J Cell Mol Med. 2025 Feb 24;29(4):e70431. doi: 10.1111/jcmm.70431 (PMC11850097; doi:10.1111/jcmm.70431)

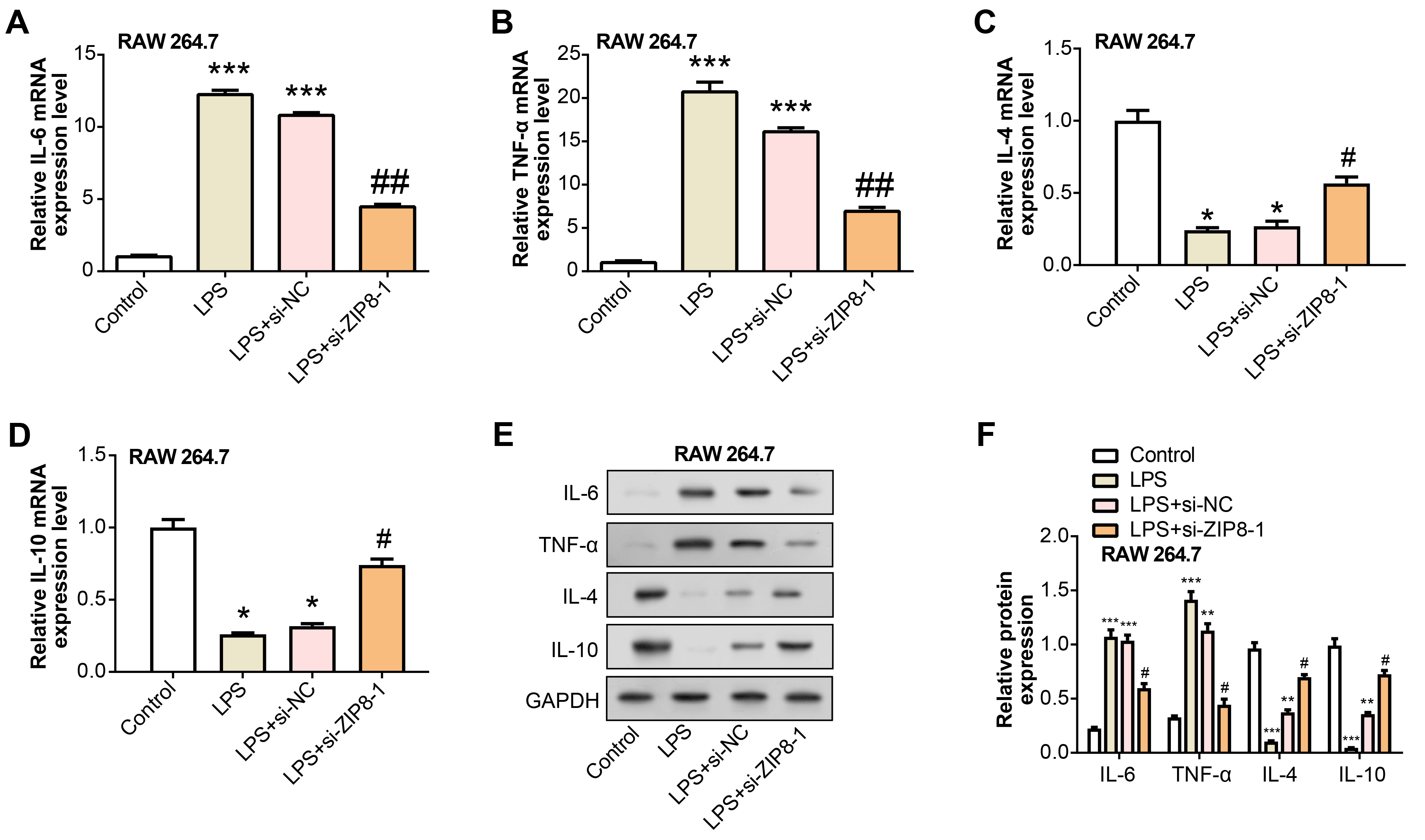

Supplement: Supplementary file 3 — Figure S3. Knockdown of ZIP8 inhibits M1 polarisation. (A–D) Relative mRNA expression levels of proinflammatory cytokines IL‐6 (A), TNF‐α (B) and anti‐inflammatory cytokines IL‐4 (C) and IL‐10 (D) in RAW 264.7 cells treated with LPS, LPS + si‐NC or LPS + si‐ZIP8‐1, as measured by qRT‐PCR. *p < 0.05, ***p < 0.001 versus control, # p < 0.05 versus LPS + si‐NC group. (E and F) WB analysis of IL‐6, TNF‐α, IL‐4 and IL‐10 protein levels in RAW 264.7 cells under the same conditions. **p < 0.01 versus control group, ***p < 0.001 versus control group, #p < 0.05, ##p < 0.01 versus LPS + si‐NC group. qRT‐PCR, quantitative real‐time polymerase chain reaction; WB, western blot; LPS, lipopolysaccharide. [file JCMM-29-e70431-s003.tif]

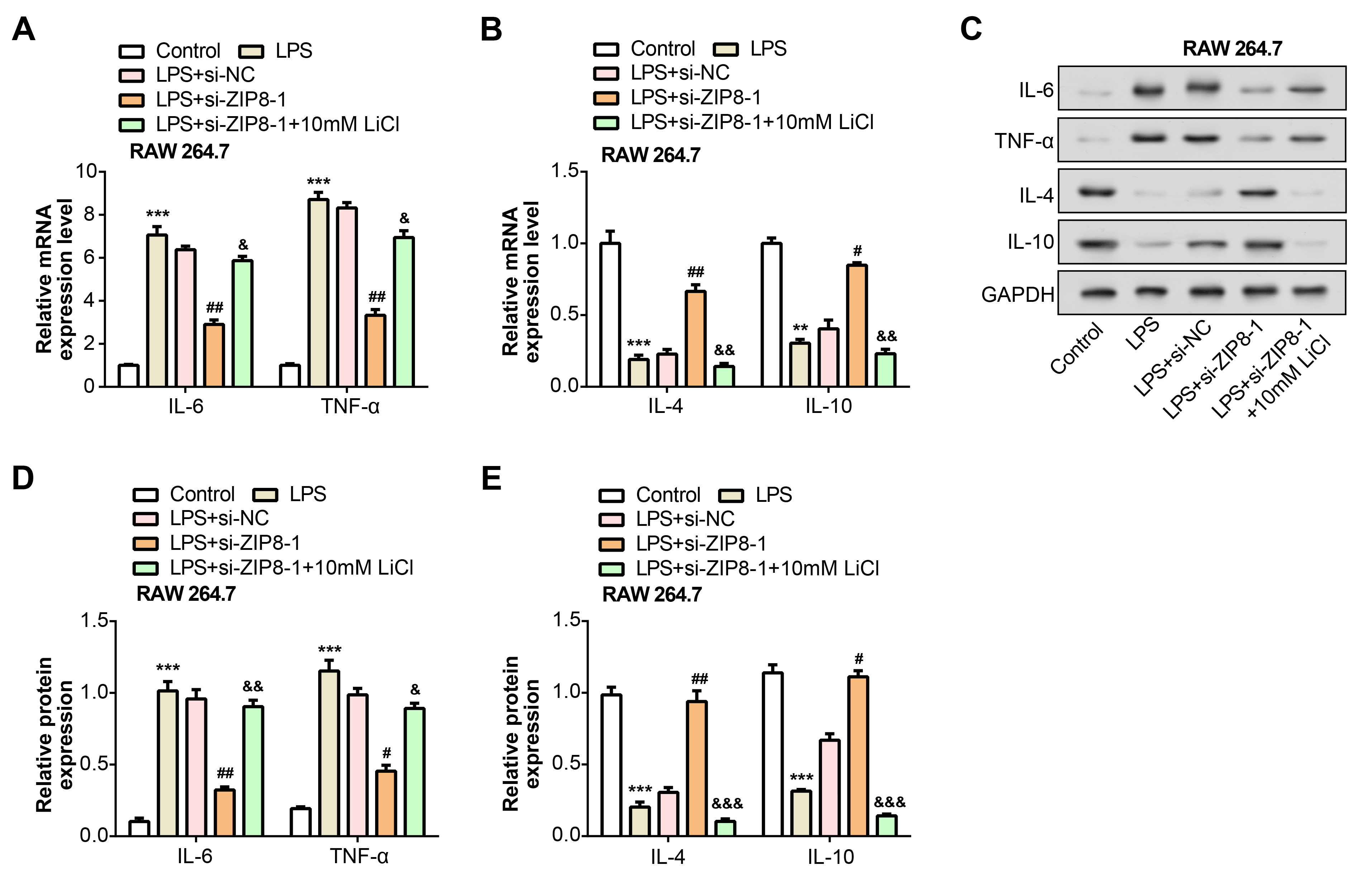

Supplement: Supplementary file 4 — Figure S4. Effects of ZIP8 knockdown and LiCl treatment on cytokine expression in RAW 264.7 cells. (A and B) Relative mRNA expression levels of M1 macrophage markers (IL‐6 and TNF‐α) (A) and M2 macrophage markers (IL‐4 and IL‐10) (B) in RAW 264.7 cells under LPS stimulation, ZIP8 knockdown (si‐ZIP8‐1) and LiCl treatment. **p < 0.01 versus control group, ***p < 0.001 versus control group, # p < 0.05 versus LPS + si‐NC group, ## p < 0.01 versus LPS + si‐NC group, & p < 0.05 vs. LPS + si‐ZIP8‐1group, && p < 0.01 vs. LPS + si‐ZIP8‐1 group. (C) WB analysis of IL‐6, TNF‐α, IL‐4 and IL‐10 in RAW 264.7 cells across the same conditions. (D and E) Quantification of the relative protein expression levels of M1 macrophage markers (IL‐6, TNF‐α) (D) and M2 macrophage markers (IL‐4, IL‐10) (E) in RAW 264.7 cells. ***p < 0.001 versus control group, # p < 0.05 versus LPS + si‐NC group, ## p < 0.01 versus LPS + si‐NC group, & p < 0.05 versus LPS + si‐ZIP8‐1 group, && p < 0.01 versus LPS + si‐ZIP8‐1 group, &&& p < 0.001 versus LPS + si‐ZIP8‐1 group. qRT‐PCR, quantitative real‐time polymerase chain reaction; WB, western blot; LPS, lipopolysaccharide. [file JCMM-29-e70431-s002.tif]
