# Supplementary material for: ZIP8 Regulates Inflammation and Macrophage Polarisation in Intervertebral Disc Degeneration via the Wnt/β‐Catenin Pathway
Source: J Cell Mol Med. 2025 Feb 24;29(4):e70431. doi: 10.1111/jcmm.70431 (PMC11850097; doi:10.1111/jcmm.70431)
Supplement: Supplementary file 5 — Table S1. Human NP (nucleus pulposus) cells’ primer sequences. [file JCMM-29-e70431-s001.docx]

**Supplementary Table 1. Human NP (Nucleus Pulposus) Cells Primer Sequences.**

| **Gene** | **Forward Primer (5' → 3')** | **Reverse Primer (5' → 3')** |
| --- | --- | --- |
| *ZIP8* | GAGTTTCACCGCGGACCTG | TAGCCGAGGGGAGCGATAG |
| *β-catenin* | GTTGAGCACCTGTTTGCCTG | GTTGAGCACCTGTTTGCCTG |
| *MMP13* | AGACCTCCAGTTTGCAGAGC | ATCAGGAACCCCGCATCTTG |
| *ADAMTS5* | GGCTCACGAAATCGGTAACTG | GGCGAGCACAGACATCCAT |
| *Aggrecan* | GACTTCCGCTGGTCAGATGG | CGTTTGTAGGTGGTGGCTGT |
| *COL II* | GCTCCTGCCGTTTCGCTG | ATTATACCTCTGCCCATCCTGC |
| *NF-κB* | GACTCGCCACCCGGCTT | GTATGGGCCATCTGTTGGCAG |
| *GAPDH* | AATGGGCAGCCGTTAGGAAA | GCGCCCAATACGACCAAATC |
