# Supplementary material for: ZIP8 Regulates Inflammation and Macrophage Polarisation in Intervertebral Disc Degeneration via the Wnt/β‐Catenin Pathway
Source: J Cell Mol Med. 2025 Feb 24;29(4):e70431. doi: 10.1111/jcmm.70431 (PMC11850097; doi:10.1111/jcmm.70431)
Supplement: Supplementary file 6 — Table S2. Rat intervertebral disc tissue primer sequences. [file JCMM-29-e70431-s004.docx]

**Supplementary Table 2. Rat Intervertebral Disc Tissue Primer Sequences.**

| **Gene** | **Forward Primer (5' → 3')** | **Reverse Primer (5' → 3')** | |
| --- | --- | --- | --- |
| *ZIP8* | TCCTTTTATCTCAGGCTCCGC | TGTCGGTGCCCTTAGGGTG | |
| *β-catenin* | GTGGAGCACGCACAGATAGG | CAGGTAAGATTGCTGCTGCC |  |
| *MMP13* | TCCATCCCGAGACCTCATGT | CCCCGTGTCCTCAAAGTGAA |  |
| *ADAMTS5* | GTGGGGAAGACAAGGCCTAC | GTGTAGGCCCAGGGATGTTC |  |
| *Aggrecan* | AGCCCTTGTCTGAATGGAGC | GGTCGGGAAAGTGGCGATAA |  |
| *COL II* | ATGTATGGAAGCCCTCGTCC | GGCCCTAATTTTCCACTGGC |  |
| *GAPDH* | GAAGGTCGGTGTGAACGGAT | ACCAGCTTCCCATTCTCAGC |  |
