# Supplementary material for: ZIP8 Regulates Inflammation and Macrophage Polarisation in Intervertebral Disc Degeneration via the Wnt/β‐Catenin Pathway
Source: J Cell Mol Med. 2025 Feb 24;29(4):e70431. doi: 10.1111/jcmm.70431 (PMC11850097; doi:10.1111/jcmm.70431)
Supplement: Supplementary file 7 — Table S3. Mouse RAW 264.7 macrophages’ primer sequences. [file JCMM-29-e70431-s005.docx]

**Supplementary Table 3. Mouse RAW 264.7 Macrophages Primer Sequences.**

| **Gene** | **Forward Primer (5' → 3')** | **Reverse Primer (5' → 3')** |
| --- | --- | --- |
| *β-catenin* | GTCAGCTCGTGTCCTGTGAA | GGCCATGTCCAACTCCATCA |
| *MMP13* | ACCCAGCCCTATCCCTTGAT | GGTCACGGGATGGATGTTCA |
| *ADAMTS5* | ACACTGAAACATGGGACCGT | ACAGAAACCATACAAGTGCCT |
| *Aggrecan* | TTGACGAGTGCCTCTCAAGC | CGGTCATGAAAGTGGCGGTA |
| *COL II* | ATGAGGGAGCGGTAGAGACC | GGCCCTAATTTTCGGGCATC |
| *C-myc* | CGACTACGACTCCGTACAGC | GTAGCGACCGCAACATAGGA |
| *IL-1β* | GCCACCTTTTGACAGTGATGAG | AGCTTCTCCACAGCCACAAT |
| *COX-2* | CATAAGCGAGGACCTGGGTT | TGGCATACATCATCAGACCA |
| *iNOS* | GGAGCGCTCTAGTGAAGCAA | TCCACTGCCCCAGTTTTTGA |
| *IL-6* | TGATGGATGCTACCAAACTGGA | TGTGACTCCAGCTTATCTCTTGG |
| *TNF-α* | CCCTCACACTCACAAACCAC | CCCTTGAAGAGAACCTGGGAG |
| *IL-4* | CAAACGTCCTCACAGCAACG | GGCATCGAAAAGCCCGAAAG |
| *IL-10* | GCTCCAAGACCAAGGTGTCT | CCAAGGAGTTGTTTCCGTTAGC |
| *GAPDH* | CCCTTAAGAGGGATGCTGCC | ATGAAGGGGTCGTTGATGGC |
